# Supplementary material for: Pre- and post-migration determinants of self-rated health among Ukrainian refugees in Germany: A cross-sectional comparative analysis with recently arrived refugees from other countries of origin
Source: PLOS Glob Public Health. 2025 Nov 10;5(11):e0004565. doi: 10.1371/journal.pgph.0004565 (PMC12599935; doi:10.1371/journal.pgph.0004565)
Supplement: S1 File — (DOCX) [file pgph.0004565.s001.docx]

**Supplementary Information (S1 File) for**

**Pre- and post-migration determinants of self-rated health among Ukrainian refugees in Germany: a cross-sectional comparative analysis with recently arrived refugees from other countries of origin**

Louise Biddle^1^, Andrea Marchitto^1^, Sabine Zinn^1,2^*

^1^ Socio-Economic Panel, German Institute for Economic Research (DIW Berlin), Berlin, Germany

^2^ Institute of Social Sciences, Humboldt University Berlin, Berlin, Germany

* szinn@diw.de

**Supplementary information**

[Supplementary Table A: Categorisation of variables used in Ukrainian and non-Ukrainian samples 3](#_Toc195002110)

[Supplementary Table B: Categorisation of additional variables used in sensitivity analyses 9](#_Toc195002111)

[Supplementary Figure A: Cumulated fatalities in Ukrainian regions between 24th February 2022 and 16th March 2022*, ACLED data 11](#_Toc195002112)

[Supplementary Table C: Regression results main analyses – Pre- and post-migration determinants of self-rated health, Ukrainian refugees 12](#_Toc195002113)

[Supplementary Table D: Variance Inflation Factors (VIFs) of variables included in full models of both samples 16](#_Toc195002114)

[Supplementary Table E: Regression results main analyses – Pre- and post-migration determinants of self-rated health, non-Ukrainian refugees 17](#_Toc195002115)

[Supplementary Table F: Regression results sensitivity analyses (SA1-SA5) – Pre- and post-migration determinants of self-rated health, Ukrainian refugees 21](#_Toc195002116)

[Supplementary Table G: Regression results sensitivity analyses (SA6) – Pre- and post-migration determinants of self-rated health with migration cohorts as an additional covariate, Ukrainian refugees 26](#_Toc195002117)

[Supplementary Table H: Regression results sensitivity analyses (SA4, SA5, SA7) – Pre- and post-migration determinants of self-rated health, non-Ukrainian refugees 30](#_Toc195002118)

[Supplementary Table J: Regression results sensitivity analyses (SA8)– Pre- and post-migration determinants of self-rated health stratified along level of educational, Ukrainian refugees 34](#_Toc195002119)

[Supplementary Table J: Regression results sensitivity analyses (SA8)– Pre- and post-migration determinants of self-rated health stratified along level of educational, non-Ukrainian refugees 37](#_Toc195002120)

[Supplementary Table K: Regression results sensitivity analyses (SA9) – Pre- and post-migration determinants of self-rated health with interaction effects between gender and perceived discrimination, gender and social isolation, between age group and perceived discrimination, and age group and social isolation, Ukrainian refugees 40](#_Toc195002121)

[Supplementary Table L: Regression results sensitivity analyses (SA9) – Pre- and post-migration determinants of self-rated health with interaction effects between gender and perceived discrimination, gender and social isolation, between age group and perceived discrimination, and age group and social isolation, non-Ukrainian refugees 44](#_Toc195002122)

# ****Table A: Categorisation of variables used in Ukrainian and non-Ukrainian samples****

| **Variable** | **Type** | **Categories** | **Description** |
| --- | --- | --- | --- |
| ***Outcome variable*** | | | |
| General health (self-rated) | Binary | [Good/satisfactory; Bad] | Self-rated general health assessment:  „*How would you describe your general state of health?* (UKR)”/ „*How would you describe your current state of health?* (non-UKR)”  Good/satisfactory: very good, good, satisfactory  Bad: bad, very bad |
|  |  |  |  |
| ***Sociodemographics*** | | | |
| Gender | Binary | [Male; Female] | Self-reported sex |
| Age group | Categorical | [18-30; 31-49; 50+] | Difference between survey year and respondents’ year of birth |
| Economic situation before flight/war | Categorical | [(well)below average; on average; (well)above average] | Self-assessment of own’s economic situation before leaving the country, or before the war in the country started, compared to the economic situation of people of the same country: „*How would you rate your financial situation before war, i.e. before February 2022, compared to the situation of others in Ukraine* (UKR)” / „*How would you estimate your financial situation at that time with the income of other people in your country?* (non-UKR)”  (well)below average: considerably below average, below average / well below average, below average  On average: average  (well)above average: considerably above average, above average / well above average, above average |
| Education | Categorical | [Low; Medium; High] | Ukraine sample:  „*Did you complete vocational training or university studies?* (UKR)”  Low: whether respondents possess [named], at least, one or more titles (low) or they have no vocational training or academic title. Respondents in this category do not possess any higher title (in categories considered as medium and high).   - I haven’t completed vocational training or academic studies - Certificate of transfer or upgrade of job qualification, up to one year of vocational training - Completion of the second year of study leading to Junior Specialist Diploma   Medium: whether respondents possess [named], at least, one or more titles (medium). Respondents in this category do not possess any higher title (high).   - Certificate/Confirmation of assignment or upgrade of job qualification, with full general secondary school leaving certificate, 2–3-years of study - Diploma in secondary vocational education, 4 years of study   High: whether respondents possess [named], at least, one or more titles (high).   - Advanced technical college certificate, 1 year of study - Advanced technical college certificate, 2–3 years of study - Academic reference - Bachelor’s degree, 4 years (based on current university system) - Master’s degree, 6 years (based on current university system) - Specialized diploma, 5–6 years - Postgraduate studies - Academic rank, doctoral candidate, Doctor of Science   Non-Ukraine sample: International Standard Classification of Education (ISCED), 2011  Low: in school, Primary education, Lower secondary education  Medium: Upper secondary education, Post-secondary non-tertiary education  High: Short-cycle tertiary education, Bachelor’s or equiv. level, Master’s or equiv. level, Doctoral or equiv. level |
| Employed before flight | Binary | [No; Yes] | Ukraine sample: „*Were you ever gainfully employed prior to moving to Germany?*”  No  Yes  Non-Ukraine sample:  *„Q1. How old were you when you worked for the first time in your life?” „Q2. What was the last job you had in your country of origin?”*  No: I have never had a job, I never had a job in my country of origin [named in Q1]  Yes: whether respondents provided an occupational activity (in Q2) and if they reported the age of first time they worked (Q1) |
| Country of birth (only non-Ukrainian refugees) | Categorical | [Syria; Afghanistan; Iraq;  Other countries] | Self-reported country of birth  Other countries: Albania, Algeria, Angola, Azerbaijan, Bangladesh, Armenia, Bosnia and Herzegovina, Myanmar, Cameroon, Sri Lanka, Costa Rica, Ethiopia, Eritrea, Georgia, Gambia, Palestine, Guinea, Iran, Ivory Coast, Kenya, Kuwait, Lebanon, Libya, Mali, Morocco, Niger, Nigeria, Pakistan, Romania, Russia, Saudi Arabia, Serbia, Vietnam, Somalia, Sudan Tajikistan, Turkey, North Macedonia, Uzbekistan, Kosovo |
|  |  |  |  |
| ***Pre-migration factors*** | | | |
| PTS | Binary | [Low (if PTS avg <=3); High (if PTS avg>3)] | PTS:  Three sources, (Amnesty International; U.S. State Department; Human Right Watch) assess a level for each country each year in the 5-level “terror scale” index.   - 1: Coun­tries un­der a se­cure rule of law, people are not im­prisoned for their views, and tor­ture is rare or ex­cep­tion­al. Polit­ic­al murders are ex­tremely rare - 2: There is a lim­ited amount of im­pris­on­ment for non­vi­ol­ent polit­ic­al activ­ity. However, few per­sons are af­fected, tor­ture and beat­ings are ex­cep­tion­al. Polit­ic­al murder is rare - 3: There is ex­tens­ive polit­ic­al im­pris­on­ment, or a re­cent his­tory of such im­pris­on­ment. Ex­e­cu­tion or oth­er polit­ic­al murders and bru­tal­ity may be com­mon. Un­lim­it. de­ten­tion, with or without a tri­al, for polit­ic­al views is ac­cep­ted - 4: Civil and polit­ic­al rights vi­ol­a­tions have ex­pan­ded to large num­bers of the pop­u­la­tion. Murders, dis­ap­pear­ances, and tor­ture are a com­mon part of life. In spite of its gen­er­al­ity, on this level ter­ror af­fects those who in­terest them­selves in polit­ics or ideas - 5: Ter­ror has ex­pan­ded to the whole pop­u­la­tion. The lead­ers of these so­ci­et­ies place no lim­its on the means or thor­ough­ness with which they pur­sue per­son­al or ideo­lo­gic­al goals   Ukraine sample (regional-level):  Low: PTS-dataset has no data available for subnational level, with exception of Ukraine for year 2022. To Ukrainian regions not under Russia occupation PTS’s sources assigned values between 1 and 3 in 2022. The avg. level of PTS was matched to respondents according to their Ukrainian region of residence and their date of leaving the country (See Fig 2, manuscript)  High: Ukrainian regions under Russia occupation have been assigned a PTS level of 5 (high). This value was assigned to respondents residing in Ukrainian occupied regions, considering the date they fled from these regions and development of Russian offensive campaign in Ukraine (See Fig 2, manuscript)  Non-Ukraine sample (country-level):  Low: PTS values between 1 and 3. Only PTS levels matching respondents’ country of birth and year of leaving the country were included  High: PTS level greater than 3. Only PTS-levels matching respondents’ country of birth and year of leaving the country were included |
| Reason for leaving the country | Binary | [No reason; One/more] | To capture traumatic experiences during refugees’ pre-migration phase we used respondents’ reasons for leaving their country:  *„In addition to the war in Ukraine, there may be other reasons for leaving the country. What reasons played a role in your decision to leave Ukraine?* (UKR)” / „*What were the main reasons that made you then leave this country? (*Non-UKR)”  Reasons for leaving the country considered for the analysis 🡪 Ukraine sample: war or violent conflict in Ukraine, persecution or discrimination (ethnic, religious, etc.), poor living condition  Non-Ukraine sample: expulsion or deportation, persecution, discrimination (ethnic, religious, etc.), poor personal living condition  No reason: if respondents named none of these reasons for leaving their country  One/more: whether respondents named one or more of these reasons that made them leave the country |
|  |  |  |  |
| ***Post-migration factors*** | | | |
| Time since arrival in Germany | Binary | [0-6 months; 6-12 months] | Difference between day of data collection and arrival date in Germany (in days) [0-365]  0-6 months: 0-182 days  6-12 months: 183-365 days |
| Type of accommodation | Categorical | [Shared accommodation for refugees; private apartment/house; Other accommodation] | Ukraine sample:  „*Which type of accommodation do you currently live in?*”  Shared accom. for refugees: in shared accommodation for refugees  Private apartment/house: in a private rented apartment/private rented house  Other accommodation: other accommodation (e.g., hotel/bed and breakfast, etc.)  Non-Ukraine sample:  „*Q1. Now, please think of the accommodation in which you were housed the longest in Germany before your current accommodation. What type of accommodation did you live in?”*  „*Q2. In what type of accommodation does the interviewee live?”*  If respondents never moved or they moved only once [lr3235=1, 2 “Number of Accommodations in Germany”] since they arrived in Germany, *Q1* was considered. If they moved more times, *Q2* was considered  Shared accom. for refugees: large shared accommodation with other refugees, small shared accommodation with individual, separate living areas with other refugees [*Q1*], in shared accommodation [*Q2*]  Private apartment/house: a house/apartment away from shared accommodation with other refugees, a house/apartment away from shared accommodation without other refugees [*Q1*], In a private apartment, a private house [*Q2*]  Other accommodation: other accommodation [*Q1*] |
| Residence/asylum status | Categorical | UKR - [No/unclear;  Residence permit (§ 24 AsylblG/ ’Fiktionsb.’);  Other residence permit]  Non-UKR  [No/awaiting outcome;  Residence permit  (temp./perm.); Other residence permit/’Duldung’] | Ukraine sample:  „*What residence permit do you currently have?*”  No/unclear: don’t know, none, I entered the country without a visa, using my biometric passport  Residence permit (§ 24 German Asylum Law/’Fiktionsbescheinigung’): temporary residence permit, residence permit under Sect. 24 for temporary asylum  Other residence permit: None, I have a Schengen visa, another residence  Non-Ukraine sample:  „*Which residence title do you currently hold? If you are not sure which residence title you hold, please check the Immigration Authority’s label in your passport?*”  No/awaiting outcome: Permission to stay pursuant to Section 55 of the German Asylum Law (asylum seekers)  Residence permit (temporary/permanent): A residence permit according to Section 25 sub-section 1 of the German Residence Act (persons entitled to asylum), a residence permit according to Section 25 sub-section 2 of the German Residence Act (persons with refugee status), a settlement permit according to Section 26 sub-section 3 of the German Residence Act, a residence permit according to Section 22 or Section 23 of the German Residence Act (admission on humanitarian grounds)  Other residence permit/’Duldung’: a temporary suspension of deportation according to section 60a of the German Residence Act (‘Duldung: tolerated stay’), a residence permit pursuant to § 23a or § 25 sub-section 3, 4 or 5 of the German Residence Act (admission on other humanitarian grounds) |
| Partner’s living place | Categorical | [No partner;  In Germany;  Ukraine (or country of origin)/abroad] | „*What country does your partner currently live in?* (Ukr-sample)”  „*Where does your spouse (or partner) live?* (Non-Ukr-sample)”  No partner: Respondent having not a spouse or a partner are coded as reference category 🡪 (Marital status: single [pukr32=1; plj0626=1], and have partner/committed in a relationship: no [pukr33=2; plj0629=2])  In Germany: in Germany [UKR]; Here in my household / in this accommodation, in this town/location but in a different household/accommodation, elsewhere in Germany [Non-UKR]  Ukraine (or country of origin)/abroad: in Ukraine, in Polen, other [UKR]; In my country of origin, elsewhere abroad [Non-UKR] |
| Children’s living place | Categorical | [No child,  One/all in Germany; One/all in Ukraine (or country of origin)/abroad/deceased] | First three children were considered for each respondent [child 1, child 2, child 3]  „*Q1. Is your child currently living in the same household as you? Q2. If your child is not living with you, what country does he/she live in?* (UKR)”  „*Where does this child currently live?* (Non-UKR)”  No child: Respondents without children are coded as reference category 🡪 (I don’t have any children [pukr39n0=1]; How many children do/did you have? [lb0285=-2 “does not apply”])  One/all in Germany: if one or all children were in Germany 🡪 In my household, in Germany [UKR]; Here in my household / in this accommodation, in this town/location but in a different household/accommodation, elsewhere in Germany [Non-UKR]  One/all in Ukraine (or country of origin)/abroad/deceased: Ukraine, elsewhere abroad [UKR]; In my country of origin, elsewhere abroad, this child is deceased [Non-UKR] |
| Perceived discrimination | Binary | [Never;  Seldomly/often] | Self-reported experience of being discriminated (disadvantaged) against because of one’s own origin  Never: never  Seldomly/often: occasionally, frequently |
| Attended German language/integration course/ESF-BAMF course | Binary | [No; Yes] | Whether respondents attended one or more German language or integration courses or ESF-BAMF courses (only for non-UKR. refugees) or not  No: respondent did not attend any course [not named]  Yes: respondent attended one or more courses [named] |
| German language proficiency | Categorical | [None/poor; Sufficient;  Good/excellent] | Three dimensions of respondents’ self-reported ability of German language were considered: ability to speak, read, and write. The original 5-points-Likert scale of all variables [1: “can’t [speak; read; write] in German”/“not at all”, 5: “very well”] was inverted and an average level out of all three language’s dimensions was calculated and cut at (MIN,2,3,4,5,MAX).  None/poor: avg. value between Min and 2 (included)  Sufficient: avg. value equal to 3  Good/excellent: avg. value between 4 (included) and Max |
| Social isolation | Binary | UKR [Does not apply/ neutral position; applies (fully)]  Non-UKR  [Never to sometimes; (very) Often] | Self-reported assessment of social isolation  Ukraine sample:  „*The following questions are about how you see yourself. To what extent do the following statements apply to you?: I feel alone*”  Does not apply/neutral position: do not apply at all, do not apply, neutral position  Applies (fully): applies, applies fully  Non-Ukraine sample:  „*How often do you feel socially isolated?*”  Never to sometimes: never, occasionally, sometimes  (very) Often: often, very often |
| Contact with Ukrainians (/persons of same country of origin) [non-relative] | Categorical | [No/seldomly;  Often;  Very often] | Self-reported assessment of contact with persons from the same country of origin [non-relatives]  „*How often do you spend time with people from Ukraine (people from your country of origin) living in Germany who are not related to you?*”  No/seldomly: never, less often/seldom  Often: every month, every week  Very often: several times per week, every day/daily |
| Contact with Germans | Categorical | [No/seldomly;  Often;  Very often] | Self-reported assessment of contact with Germans  „*How often do you spend time with Germans?*”  No/seldomly: never, less often/seldom  Often: every month, every week  Very often: several times per week, every day/daily |
|  |  |  |  |

UKR: Sample of Ukrainian refugees; non-UKR: Sample of refugees from other countries of origin; PTS: Political Terror Scale

# ****Table B: Categorisation of additional variables used in sensitivity analyses****

| **Variable** | **Type** | **Categories** | **Description** |
| --- | --- | --- | --- |
| **Outcome variable** | | | |
| General health (self-rated) | Binary | [Good;  Satisfactory/bad] | Self-rated general health assessment:  „*How would you describe your general state of health?* (UKR)”/ „*How would you describe your current state of health?* (non-UKR)”  Good: very good, good  Satisfactory/bad: satisfactory, bad, very bad |
| Concerned about health situation [only UKR] | Binary | [Not at all/little concerned; very concerned] | Self-assessment of being concerned (being worried about) of one’s own health situation  Not at all/little concerned: no, no worries, yes, some worries  Very concerned: yes, big worries |
|  |  |  |  |
| **Pre-migration factors** | | | |
| Fatalities (number of deaths) between war’s start and date of leaving Ukraine  [only UKR] | Categorical [in quintiles] | [1Q: 0-10; 2Q: 12-54;  3Q: 58-160; 4Q: 162-720; 5Q: 732-2532] | We used the Armed Conflict Location & Data Event Data Project – ACLED Conflict Index^1,2^, collecting information on dates, actors, locations, number of fatalities and political violence/protest events around the world, to investigate whether the intensity of war, captured by counting and cumulating the number of fatalities (number of deaths) by region in Ukraine in the pre-migration phase affects our outcomes variable.  ACLED Data were pre-prepared to be integrated with IAB-BiB/FReDA-BAMF-SOEP Survey dataset with the following steps:   1. variable ***event_date*** was destringed and recoded to be compatible with STATA date format mdy (%td) 2. observations were considered if ***country***= “Ukraine”, ***civilian_targeting***= ”Civilian targeting”, and by the most accurate time precision records: ***time_precision***=1 3. variable ***admin1***, indicating Ukrainian regions, was recoded for matching the corresponding variable in IAB-BiB/FReDA-BAMF-SOEP Survey dataset 4. only variables ***fatalities***, ***admin1*** (recoded), ***event_date*** (recoded) were taken before merging datasets^3^ 5. According to ACLED data, it is possible to experience missing dates in dataset (***event_date***) because no event (***fatalities***), occurred, or to experience multiple observations (***fatalities***) each date, because more events occurred in different places within regions, or, at different times. We manually filled up missing dates (continuous dates) by imputing value “*0*”. Successively, we cumulated fatalities by ***event_date*** and Ukrainian region (***admin1***, recoded), to have only one observation of fatalities for each date and region 6. ACLED (modified) and IAB-BiB/FReDA-BAMF-SOEP Survey datasets were finally merged   We cumulated all occurred fatalities, by respondent and his/her region of residence in Ukraine, between the day the war started (24th February 2022) and respondent’s own date of leaving the country. Observations were split into five quantiles:  1Q: 0-10 2Q: 12-54  3Q: 58-160 4Q: 162-720 5Q: 732-2532 |
|  |  |  |  |
| ***Post-migration factors*** | | | |
| Fatalities (number of deaths) until 7 days before day of data collection [only UKR] | Categorical [in tertiles] | [1Q: 0;  2Q: 2-4; 3Q: 6-94] | We used ACLED Data^1^, to investigate whether the intensity of war, captured by counting and cumulating the number of fatalities (number of deaths) by region in Ukraine in the post-migration phase affects our outcomes variable.  ACLED Data were pre-prepared to be integrated with IAB-BiB/FReDA-BAMF-SOEP Survey dataset following the steps 1-6 (as for “Fatalities” in pre-migration phase)  We cumulated all occurred fatalities by respondent and his/her region of residence in Ukraine, from the day respondents concluded the survey questionnaire (included) until seven days before. Observations were split into three quantiles:  1Q: 0  2Q: 2-4 3Q: 6-94 |
|  |  |  |  |

UKR: Sample of Ukrainian refugees; non-UKR: Sample of refugees from other countries of origin; ACLED: Armed Conflict Location & Data Event Data

Figure A: Cumulated fatalities in Ukrainian regions between 24th February 2022 and 16th March 2022*, ACLED data^1^


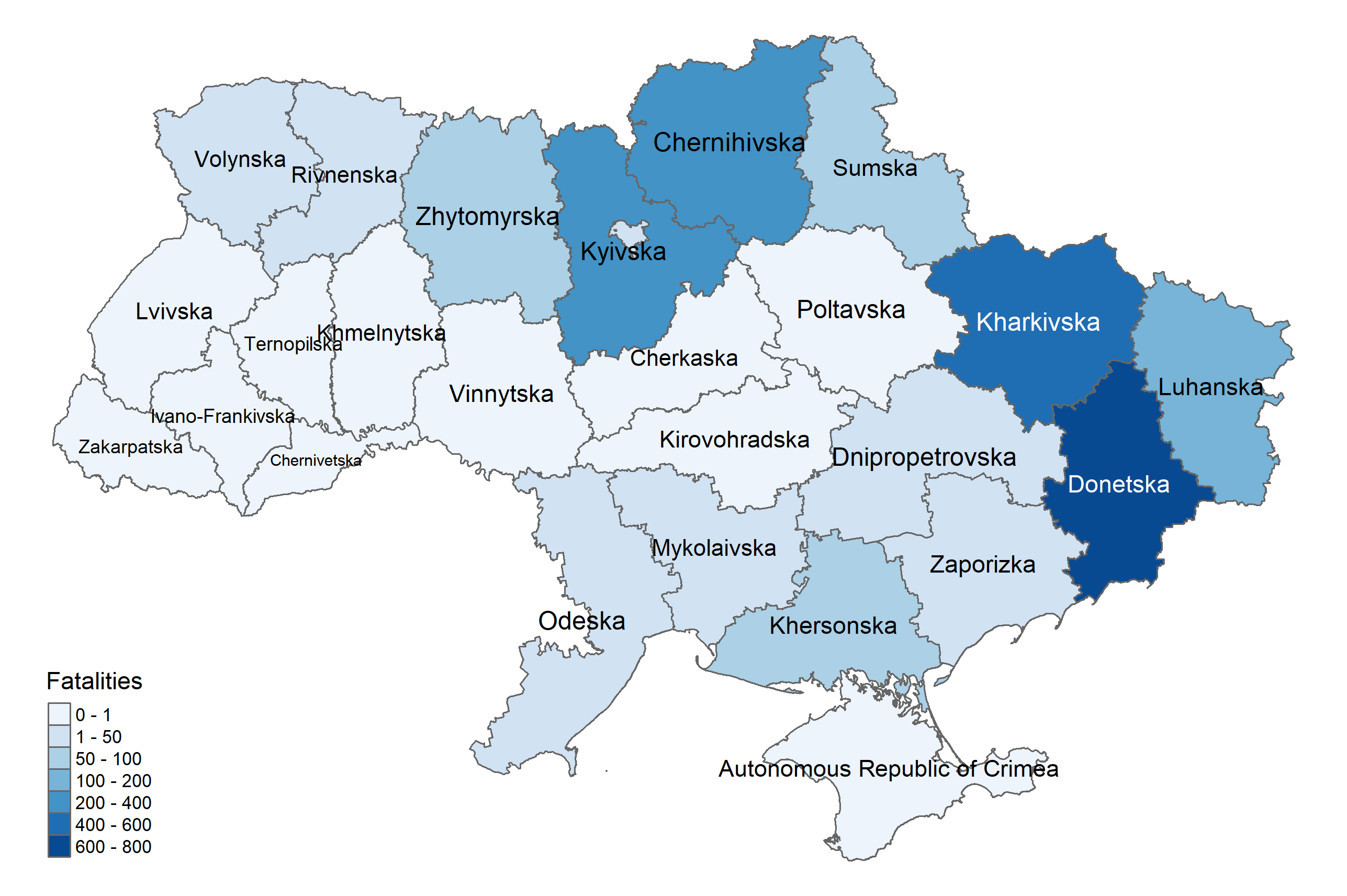


* Arithmetic mean date of leaving Ukraine in the Ukrainian refugee sample

# Table C: Regression results main analyses – Pre- and post-migration determinants of self-rated health, Ukrainian refugees

|  | **Self-rated health** | | | |
| --- | --- | --- | --- | --- |
|  | **M1** | **M2** | **M3** | **M4** |
|  | **Socio-demographics** | **Socio-demographics**  **+**  **pre-migration factors** | **Socio-demographics**  **+**  **post-migration factors** | **Fully adjusted model** |
|  |  |  |  |  |
| SOCIODEMOGRAPHICS | | | | |
|  |  |  |  |  |
| **Gender** |  |  |  |  |
| r. male |  |  |  |  |
|  |  |  |  |  |
| female | 1.058 | 1.063 | 0.948 | 0.951 |
|  | [0.850,1.317] | [0.854,1.323] | [0.743,1.209] | [0.745,1.212] |
|  |  |  |  |  |
| **Age group** |  |  |  |  |
| r. 18-30 |  |  |  |  |
|  |  |  |  |  |
| 31-49 | 0.901 | 0.903 | 1.004 | 1.001 |
|  | [0.715,1.137] | [0.716,1.139] | [0.747,1.351] | [0.744,1.346] |
|  |  |  |  |  |
| 50+ | **1.665^***^** | **1.670^***^** | **2.064^***^** | **2.068^***^** |
|  | [1.308,2.121] | [1.311,2.127] | [1.466,2.907] | [1.468,2.912] |
|  |  |  |  |  |
| **Economic situation before flight** |  |  |  |  |
| r. (well)below average |  |  |  |  |
|  |  |  |  |  |
| on average | **0.698^***^** | **0.700^**^** | **0.766^*^** | **0.767^*^** |
|  | [0.564,0.864] | [0.566,0.867] | [0.613,0.958] | [0.613,0.959] |
|  |  |  |  |  |
| (well)above average | **0.620^***^** | **0.623^***^** | **0.627^***^** | **0.630^***^** |
|  | [0.479,0.803] | [0.481,0.807] | [0.479,0.821] | [0.481,0.825] |
|  |  |  |  |  |
| **Level of education** |  |  |  |  |
| r. low |  |  |  |  |
|  |  |  |  |  |
| medium | 0.999 | 0.997 | 1.003 | 0.999 |
|  | [0.738,1.352] | [0.737,1.349] | [0.732,1.373] | [0.729,1.368] |
|  |  |  |  |  |
| high | **0.736^*^** | **0.739^*^** | 0.777 | 0.779 |
|  | [0.577,0.941] | [0.579,0.944] | [0.602,1.002] | [0.604,1.005] |
|  |  |  |  |  |
| **Employed before flight** |  |  |  |  |
| r. no |  |  |  |  |
|  |  |  |  |  |
| yes | **0.723^**^** | **0.720^**^** | **0.746^*^** | **0.743^*^** |
|  | [0.581,0.901] | [0.578,0.897] | [0.593,0.939] | [0.590,0.935] |
|  |  |  |  |  |
| PRE-MIGRATION FACTORS | | | | |
|  |  |  |  |  |
| **PTS (avg)** |  |  |  |  |
| r. low (<=3) |  |  |  |  |
|  |  |  |  |  |
| high (>3) |  | 1.009 |  | 0.986 |
|  |  | [0.850,1.199] |  | [0.825,1.178] |
|  |  |  |  |  |
| **Reason for leaving the country** |  |  |  |  |
| r. other reasons |  |  |  |  |
|  |  |  |  |  |
| war, conflict and/or persecution |  | 0.834 |  | 0.826 |
|  |  | [0.575,1.209] |  | [0.560,1.216] |
|  |  |  |  |  |
| POST-MIGRATION FACTORS | | | | |
|  |  |  |  |  |
| **Time in Germany since arrival** |  |  |  |  |
| r. 0-6 months |  |  |  |  |
|  |  |  |  |  |
| 6-12 months |  |  | 0.799 | 0.801 |
|  |  |  | [0.555,1.151] | [0.556,1.153] |
|  |  |  |  |  |
| **Type of accomodation** |  |  |  |  |
| r. shared accom. for refugees |  |  |  |  |
|  |  |  |  |  |
| private apartment/house |  |  | 0.863 | 0.862 |
|  |  |  | [0.628,1.185] | [0.628,1.184] |
|  |  |  |  |  |
| other accommodation |  |  | 0.974 | 0.972 |
|  |  |  | [0.671,1.412] | [0.670,1.410] |
|  |  |  |  |  |
| **Residence/asylum status** |  |  |  |  |
| r. no/unclear |  |  |  |  |
|  |  |  |  |  |
| other resid. permits |  |  | 0.942 | 0.928 |
|  |  |  | [0.433,2.048] | [0.426,2.021] |
|  |  |  |  |  |
| resid. Permit (TPD, §24 AsylG) |  |  | 1.154 | 1.162 |
|  |  |  | [0.639,2.082] | [0.643,2.098] |
|  |  |  |  |  |
| **Partner's living place** |  |  |  |  |
| r. no partner |  |  |  |  |
|  |  |  |  |  |
| Germany |  |  | 1.010 | 1.010 |
|  |  |  | [0.809,1.262] | [0.809,1.262] |
|  |  |  |  |  |
| Ukraine/abroad |  |  | 0.806 | 0.808 |
|  |  |  | [0.636,1.021] | [0.637,1.024] |
|  |  |  |  |  |
| **Children living place** |  |  |  |  |
| r. no child |  |  |  |  |
|  |  |  |  |  |
| one/all in Germany |  |  | 0.873 | 0.879 |
|  |  |  | [0.665,1.146] | [0.669,1.154] |
|  |  |  |  |  |
| one/all in Ukraine/abroad |  |  | 0.986 | 0.986 |
|  |  |  | [0.703,1.383] | [0.703,1.383] |
|  |  |  |  |  |
| **Perceived discrimination** |  |  |  |  |
| r. never |  |  |  |  |
|  |  |  |  |  |
| seldomly/often |  |  | **1.879^***^** | **1.879^***^** |
|  |  |  | [1.559,2.264] | [1.559,2.265] |
|  |  |  |  |  |
| **Attended German language/integration course** |  |  |  |  |
| r. no |  |  |  |  |
|  |  |  |  |  |
| yes |  |  | **0.716^**^** | **0.718^**^** |
|  |  |  | [0.585,0.876] | [0.587,0.878] |
|  |  |  |  |  |
| **German language proficiency** |  |  |  |  |
| r. none/poor |  |  |  |  |
|  |  |  |  |  |
| sufficient |  |  | **0.732^*^** | **0.731^*^** |
|  |  |  | [0.554,0.968] | [0.553,0.966] |
|  |  |  |  |  |
| good/excellent |  |  | **0.370^**^** | **0.368^**^** |
|  |  |  | [0.203,0.674] | [0.202,0.672] |
|  |  |  |  |  |
| **Feeling socially isolated** |  |  |  |  |
| r. does not apply (at all)/neutral pos. |  |  |  |  |
|  |  |  |  |  |
| applies (fully) |  |  | **2.689^***^** | **2.690^***^** |
|  |  |  | [2.228,3.247] | [2.228,3.248] |
|  |  |  |  |  |
| **Contact with Ukrainians(non-relative)** |  |  |  |  |
| r. no/seldomly |  |  |  |  |
|  |  |  |  |  |
| often |  |  | **0.721^*^** | **0.723^*^** |
|  |  |  | [0.559,0.931] | [0.560,0.933] |
|  |  |  |  |  |
| very often |  |  | **0.769^*^** | **0.770^*^** |
|  |  |  | [0.613,0.964] | [0.613,0.965] |
|  |  |  |  |  |
| **Contact with Germans** |  |  |  |  |
| r. no/seldomly |  |  |  |  |
|  |  |  |  |  |
| often |  |  | 0.921 | 0.922 |
|  |  |  | [0.731,1.160] | [0.732,1.161] |
|  |  |  |  |  |
| very often |  |  | **0.670^***^** | **0.670^***^** |
|  |  |  | [0.539,0.834] | [0.538,0.833] |
|  |  |  |  |  |
| **Observations** | 5932 | 5932 | 5932 | 5932 |
| **Average degrees of freedom** | 16500000 | 24400000000 | 12100000 | 17000000 |
| **Model F-value** | 12.78 | 10.31 | 13.28 | 12.37 |
| **Model p-value (F-test)** | 0.0000 | 0.0000 | 0.0000 | 0.0000 |
| **Largest FMI** | 0.0026 | 0.0026 | 0.0348 | 0.0350 |
| Point estimates represent odds rations from multiple logistic regression models. Number in brackets represent 95% confidence intervals. Data are multiply imputed using 30 iterations.  ^*^ *p* < 0.05, ^**^ *p* < 0.01, ^***^ *p* < 0.001  Abbreviations: PTS-Political Terror Scale; TPD-Temporary Protection Directive; FMI-Fraction of Missing Information | | | | |

# Table D: Variance Inflation Factors (VIFs) of variables included in full models of both samples

|  | **Ukrainian sample (M4)**  ***VIFs*** | **Non-Ukrainian sample (M4)**  ***VIFs*** |
| --- | --- | --- |
| Gender | 1.05 | 1.49 |
| Age group | 1.82 | 1.49 |
| Economic situation before flight | 1.05 | 1.09 |
| Level of education | 1.11 | 1.16 |
| Employed before flight | 1.08 | 1.48 |
| Country of birth | - | 1.28 |
| Political Terror Scale | 1.01 | 1.12 |
| Reason for leaving the country | 1.02 | 1.04 |
| Time since arrival in Germany | 1.01 | 1.07 |
| Type of accommodation | 1.01 | 1.14 |
| Residence/ asylum status | 1.02 | 1.17 |
| Partner’s living place | 1.06 | 1.49 |
| Children’s living place | 1.72 | 1.77 |
| Perceived discrimination | 1.08 | 1.06 |
| Attended German language/ integration course | 1.09 | 1.21 |
| Feeling socially isolated (non-UKR)/ lonely (UKR) | 1.14 | 1.05 |
| Contact with people from country of origin | 1.07 | 1.14 |
| Contact with Germans | 1.11 | 1.16 |

UKR: Sample of Ukrainian refugees; non-UKR: Sample of refugees from other countries of origin

# Table E: Regression results main analyses – Pre- and post-migration determinants of self-rated health, non-Ukrainian refugees

|  | **Self-rated health** | | | |
| --- | --- | --- | --- | --- |
|  | **M1** | **M2** | **M3** | **M4** |
|  | **Socio-demographics** | **Socio-demographics**  **+**  **pre-migration factors** | **Socio-demographics**  **+**  **post-migration factors** | **Fully adjusted model** |
|  |  |  |  |  |
| SOCIODEMOGRAPHICS | | | | |
|  |  |  |  |  |
| **Gender** |  |  |  |  |
| r. male |  |  |  |  |
|  |  |  |  |  |
| female | **1.679^*^** | **1.647^*^** | **1.893^**^** | **1.882^**^** |
|  | [1.117,2.524] | [1.095,2.479] | [1.222,2.931] | [1.214,2.919] |
|  |  |  |  |  |
| **Age group** |  |  |  |  |
| r. 18-30 |  |  |  |  |
|  |  |  |  |  |
| 31-49 | **1.828^**^** | **1.770^**^** | **2.044^**^** | **1.986^**^** |
|  | [1.220,2.737] | [1.179,2.657] | [1.290,3.237] | [1.251,3.152] |
|  |  |  |  |  |
| 50+ | **4.508^***^** | **4.326^***^** | **4.280^***^** | **4.104^***^** |
|  | [2.597,7.828] | [2.480,7.545] | [2.221,8.246] | [2.119,7.947] |
|  |  |  |  |  |
| **Economic situation before flight** |  |  |  |  |
| r. (well)below average |  |  |  |  |
|  |  |  |  |  |
| on average | 0.686 | 0.688 | 0.722 | 0.720 |
|  | [0.443,1.062] | [0.444,1.067] | [0.460,1.134] | [0.458,1.132] |
|  |  |  |  |  |
| (well)above average | 0.782 | 0.785 | 0.853 | 0.848 |
|  | [0.476,1.284] | [0.477,1.292] | [0.511,1.424] | [0.507,1.419] |
|  |  |  |  |  |
| **Level of education** |  |  |  |  |
| r. low |  |  |  |  |
|  |  |  |  |  |
| medium | **0.578^*^** | **0.574^*^** | 0.619 | 0.616 |
|  | [0.348,0.960] | [0.345,0.955] | [0.367,1.043] | [0.365,1.040] |
|  |  |  |  |  |
| high | 1.045 | 1.042 | 1.246 | 1.238 |
|  | [0.648,1.686] | [0.645,1.684] | [0.751,2.067] | [0.745,2.059] |
|  |  |  |  |  |
| **Employed before flight** |  |  |  |  |
| r. no |  |  |  |  |
|  |  |  |  |  |
| yes | 0.888 | 0.885 | 0.925 | 0.915 |
|  | [0.570,1.383] | [0.567,1.380] | [0.586,1.460] | [0.580,1.445] |
|  |  |  |  |  |
| **Country of birth** |  |  |  |  |
| r. Syria |  |  |  |  |
|  |  |  |  |  |
| Afghanistan | 0.900 | 0.900 | 0.928 | 0.928 |
|  | [0.464,1.745] | [0.464,1.746] | [0.464,1.856] | [0.463,1.859] |
|  |  |  |  |  |
| Iraq | 1.461 | 1.447 | 1.386 | 1.376 |
|  | [0.919,2.323] | [0.909,2.303] | [0.837,2.297] | [0.829,2.282] |
|  |  |  |  |  |
| Other countries | 1.417 | 1.256 | 1.132 | 0.990 |
|  | [0.885,2.268] | [0.746,2.115] | [0.679,1.887] | [0.564,1.739] |
|  |  |  |  |  |
| PRE-MIGRATION FACTORS | | | | |
|  |  |  |  |  |
| **PTS** |  |  |  |  |
| r. low (<=3) |  |  |  |  |
|  |  |  |  |  |
| high (>3) |  | 0.560 |  | 0.514 |
|  |  | [0.258,1.216] |  | [0.227,1.164] |
|  |  |  |  |  |
| **Reason for leaving the country** |  |  |  |  |
| r. other reasons |  |  |  |  |
|  |  |  |  |  |
| war, conflict and/or persecution |  | 0.749 |  | 0.733 |
|  |  | [0.473,1.186] |  | [0.457,1.176] |
|  |  |  |  |  |
| POST-MIGRATION FACTORS | | | | |
|  |  |  |  |  |
| **Time in Germany since arrival** |  |  |  |  |
| r. 0-6 months |  |  |  |  |
|  |  |  |  |  |
| 6-12 months |  |  | 1.363 | 1.328 |
|  |  |  | [0.632,2.939] | [0.614,2.876] |
|  |  |  |  |  |
| **Type of accommodation** |  |  |  |  |
| r. shared accom. for refugees |  |  |  |  |
|  |  |  |  |  |
| private apartment/house |  |  | 0.661 | 0.668 |
|  |  |  | [0.433,1.009] | [0.438,1.021] |
|  |  |  |  |  |
| other accommodation |  |  | 1.191 | 1.228 |
|  |  |  | [0.258,5.496] | [0.265,5.691] |
|  |  |  |  |  |
| **Residence/asylum status** |  |  |  |  |
| r. no/awaiting outcome |  |  |  |  |
|  |  |  |  |  |
| other residence permit/'Duldung' |  |  | 1.130 | 1.087 |
|  |  |  | [0.645,1.978] | [0.616,1.916] |
|  |  |  |  |  |
| residence permit (temporary/permanent) |  |  | 1.058 | 1.048 |
|  |  |  | [0.687,1.630] | [0.678,1.618] |
|  |  |  |  |  |
| **Partner's living place** |  |  |  |  |
| r. no partner |  |  |  |  |
|  |  |  |  |  |
| Germany |  |  | 0.744 | 0.747 |
|  |  |  | [0.424,1.304] | [0.425,1.315] |
|  |  |  |  |  |
| country of origin/abroad |  |  | 1.109 | 1.136 |
|  |  |  | [0.583,2.111] | [0.594,2.172] |
|  |  |  |  |  |
| **Children living place** |  |  |  |  |
| r. no child |  |  |  |  |
|  |  |  |  |  |
| one/all in Germany |  |  | 0.936 | 0.922 |
|  |  |  | [0.541,1.618] | [0.533,1.596] |
|  |  |  |  |  |
| one/all abroad/died |  |  | 1.111 | 1.120 |
|  |  |  | [0.579,2.132] | [0.581,2.158] |
|  |  |  |  |  |
| **Perceived discrimination** |  |  |  |  |
| r. never |  |  |  |  |
|  |  |  |  |  |
| seldomly/often |  |  | 1.014 | 1.003 |
|  |  |  | [0.684,1.503] | [0.675,1.490] |
|  |  |  |  |  |
| **Attended German language/integration course** |  |  |  |  |
| r. no |  |  |  |  |
|  |  |  |  |  |
| yes |  |  | 0.773 | 0.806 |
|  |  |  | [0.523,1.141] | [0.545,1.194] |
|  |  |  |  |  |
| **German language proficiency** |  |  |  |  |
| r. none/poor |  |  |  |  |
|  |  |  |  |  |
| sufficient |  |  | 0.913 | 0.923 |
|  |  |  | [0.561,1.488] | [0.566,1.505] |
|  |  |  |  |  |
| good/excellent |  |  | 0.914 | 0.898 |
|  |  |  | [0.364,2.299] | [0.357,2.260] |
|  |  |  |  |  |
| **Feeling socially isolated** |  |  |  |  |
| r. never/sometimes |  |  |  |  |
|  |  |  |  |  |
| (very)often |  |  | **2.124^***^** | **2.161^***^** |
|  |  |  | [1.425,3.165] | [1.446,3.230] |
|  |  |  |  |  |
| **Contact with persons from country of origin (non-relative)** |  |  |  |  |
| r. no/seldomly |  |  |  |  |
|  |  |  |  |  |
| often |  |  | 1.119 | 1.181 |
|  |  |  | [0.662,1.890] | [0.695,2.004] |
|  |  |  |  |  |
| very often |  |  | 0.989 | 1.026 |
|  |  |  | [0.641,1.527] | [0.662,1.589] |
|  |  |  |  |  |
| **Contact with Germans** |  |  |  |  |
| r. no/seldomly |  |  |  |  |
|  |  |  |  |  |
| often |  |  | 0.997 | 0.989 |
|  |  |  | [0.613,1.621] | [0.606,1.613] |
|  |  |  |  |  |
| very often |  |  | 0.985 | 0.969 |
|  |  |  | [0.636,1.526] | [0.624,1.505] |
|  |  |  |  |  |
| **Observations** | 1192 | 1192 | 1192 | 1192 |
| **Average degrees of freedom** | 50500000 | 124000000 | 4352014.77 | 4839699.61 |
| **Model F-value** | 4.56 | 4.11 | 2.58 | 2.50 |
| **Model p-value (F-test)** | 0.0000 | 0.0000 | 0.0000 | 0.0000 |
| **Largest FMI** | 0.0589 | 0.0592 | 0.0612 | 0.0613 |
| Point estimates represent odds rations from multiple logistic regression models. Number in brackets represent 95% confidence intervals. Data are multiply imputed using 30 iterations.  ^*^ *p* < 0.05, ^**^ *p* < 0.01, ^***^ *p* < 0.001  Abbreviations: PTS-Political Terror Scale; FMI-Fraction of Missing Information | | | | |

# Table F: Regression results sensitivity analyses (SA1-SA5) – Pre- and post-migration determinants of self-rated health, Ukrainian refugees

|  | **General health  (self-rated)^a^** | |  | **Worries about health^b^** |  | **General health (self-rated)^c^** |  | **General health (self-rated)^a^** |
| --- | --- | --- | --- | --- | --- | --- | --- | --- |
|  | **SA1_Ukr_** | **SA2_Ukr_** |  | **SA3_Ukr_** |  | **SA4_Ukr_** |  | **SA5_Ukr_** |
|  | fully adj. model  (with ACLED pre-migration) | fully adj. model  (with PTS pre-migration + ACLED post-migration) |  | fully adj. model (main model) |  | fully adj. model (main model) |  | fully adj. model (main model. Data not imputed) |
|  |  |  |  |  |  |  |  |  |
| SOCIODEMOGRAPHICS | | | | | | | | |
|  |  |  |  |  |  |  |  |  |
| **Gender** |  |  |  |  |  |  |  |  |
| r. male |  |  |  |  |  |  |  |  |
|  |  |  |  |  |  |  |  |  |
| female | 0.951 | 0.955 |  | 0.873 |  | **1.535^***^** |  | 0.963 |
|  | [0.745,1.214] | [0.749,1.218] |  | [0.697,1.093] |  | [1.313,1.795] |  | [0.750,1.235] |
|  |  |  |  |  |  |  |  |  |
| **Age group** |  |  |  |  |  |  |  |  |
| r. 18-30 |  |  |  |  |  |  |  |  |
|  |  |  |  |  |  |  |  |  |
| 31-49 | 0.996 | 1.001 |  | 1.155 |  | 0.967 |  | 0.940 |
|  | [0.741,1.341] | [0.744,1.348] |  | [0.867,1.539] |  | [0.806,1.159] |  | [0.696,1.270] |
|  |  |  |  |  |  |  |  |  |
| 50+ | **2.036^***^** | **2.086^***^** |  | **2.759^***^** |  | **3.329^***^** |  | **1.994^***^** |
|  | [1.444,2.870] | [1.480,2.939] |  | [1.986,3.833] |  | [2.640,4.199] |  | [1.407,2.825] |
|  |  |  |  |  |  |  |  |  |
| **Economic situation before flight** |  |  |  |  |  |  |  |  |
| r. (well)below average |  |  |  |  |  |  |  |  |
|  |  |  |  |  |  |  |  |  |
| on average | **0.770^*^** | **0.771^*^** |  | **0.755^*^** |  | **0.844^*^** |  | **0.768^*^** |
|  | [0.615,0.963] | [0.616,0.964] |  | [0.608,0.938] |  | [0.719,0.992] |  | [0.611,0.966] |
|  |  |  |  |  |  |  |  |  |
| (well)above average | **0.629^***^** | **0.632^***^** |  | **0.744^*^** |  | **0.764^**^** |  | **0.636^**^** |
|  | [0.480,0.824] | [0.482,0.828] |  | [0.578,0.958] |  | [0.639,0.913] |  | [0.482,0.838] |
|  |  |  |  |  |  |  |  |  |
| **Level of education** |  |  |  |  |  |  |  |  |
| r. low |  |  |  |  |  |  |  |  |
|  |  |  |  |  |  |  |  |  |
| medium | 1.005 | 0.999 |  | 1.066 |  | 0.836 |  | 0.969 |
|  | [0.734,1.377] | [0.729,1.369] |  | [0.779,1.459] |  | [0.665,1.050] |  | [0.702,1.338] |
|  |  |  |  |  |  |  |  |  |
| high | 0.780 | 0.784 |  | 0.909 |  | **0.703^***^** |  | 0.778 |
|  | [0.604,1.006] | [0.608,1.011] |  | [0.705,1.172] |  | [0.591,0.838] |  | [0.600,1.009] |
|  |  |  |  |  |  |  |  |  |
| **Employed before flight** |  |  |  |  |  |  |  |  |
| r. no |  |  |  |  |  |  |  |  |
|  |  |  |  |  |  |  |  |  |
| yes | **0.741^*^** | **0.739^*^** |  | 1.081 |  | 1.003 |  | **0.734^*^** |
|  | [0.589,0.934] | [0.587,0.931] |  | [0.852,1.372] |  | [0.851,1.183] |  | [0.580,0.929] |
|  |  |  |  |  |  |  |  |  |
| PRE-MIGRATION FACTORS | | | | | | | | |
|  |  |  |  |  |  |  |  |  |
| **Fatalities between war's start and date of leaving Ukraine (quintiles)** |  |  |  |  |  |  |  |  |
| r. 1Q: 0-10 fatalities |  |  |  |  |  |  |  |  |
|  |  |  |  |  |  |  |  |  |
| 2Q: 12-54 fatalities | 1.148 |  |  |  |  |  |  |  |
|  | [0.905,1.456] |  |  |  |  |  |  |  |
|  |  |  |  |  |  |  |  |  |
| 3Q: 58-160 fatalities | 0.950 |  |  |  |  |  |  |  |
|  | [0.712,1.266] |  |  |  |  |  |  |  |
|  |  |  |  |  |  |  |  |  |
| 4Q: 162-720 fatalities | 1.100 |  |  |  |  |  |  |  |
|  | [0.855,1.414] |  |  |  |  |  |  |  |
|  |  |  |  |  |  |  |  |  |
| 5Q: 722-2532 fatalities | 1.145 |  |  |  |  |  |  |  |
|  | [0.812,1.613] |  |  |  |  |  |  |  |
|  |  |  |  |  |  |  |  |  |
| **PTS (avg)** |  |  |  |  |  |  |  |  |
| r. low (<=3) |  |  |  |  |  |  |  |  |
|  |  |  |  |  |  |  |  |  |
| high (>3) |  | 1.017 |  | 0.983 |  | **1.273^***^** |  | 1.010 |
|  |  | [0.821,1.261] |  | [0.831,1.164] |  | [1.137,1.425] |  | [0.842,1.211] |
|  |  |  |  |  |  |  |  |  |
| **Reason for leaving the country** |  |  |  |  |  |  |  |  |
| r. other reasons |  |  |  |  |  |  |  |  |
|  |  |  |  |  |  |  |  |  |
| war, conflict and/or persecution | 0.817 | 0.831 |  | 1.051 |  | 0.778 |  | 0.824 |
|  | [0.555,1.204] | [0.564,1.225] |  | [0.710,1.554] |  | [0.595,1.019] |  | [0.553,1.228] |
|  |  |  |  |  |  |  |  |  |
| POST-MIGRATION FACTORS | | | | | | | | |
|  |  |  |  |  |  |  |  |  |
| **Fatalities until 7 days before day of interview (tertiles)** |  |  |  |  |  |  |  |  |
| r. 1Q: 0 fatality |  |  |  |  |  |  |  |  |
|  |  |  |  |  |  |  |  |  |
| 2Q: 2-4fatalities |  | 0.806 |  |  |  |  |  |  |
|  |  | [0.599,1.086] |  |  |  |  |  |  |
|  |  |  |  |  |  |  |  |  |
| 3Q: 6-94 fatalities |  | 0.995 |  |  |  |  |  |  |
|  |  | [0.785,1.261] |  |  |  |  |  |  |
|  |  |  |  |  |  |  |  |  |
| **Time in Germany since arrival** |  |  |  |  |  |  |  |  |
| r. 0-6 months |  |  |  |  |  |  |  |  |
|  |  |  |  |  |  |  |  |  |
| 6-12 months | 0.796 | 0.807 |  | 0.850 |  | 1.164 |  | 0.772 |
|  | [0.553,1.147] | [0.560,1.162] |  | [0.596,1.214] |  | [0.910,1.488] |  | [0.533,1.119] |
|  |  |  |  |  |  |  |  |  |
| **Type of accommodation** |  |  |  |  |  |  |  |  |
| r. shared accom. for refugees |  |  |  |  |  |  |  |  |
|  |  |  |  |  |  |  |  |  |
| private apartment/house | 0.862 | 0.859 |  | 0.914 |  | 0.895 |  | 0.861 |
|  | [0.627,1.184] | [0.626,1.181] |  | [0.670,1.245] |  | [0.717,1.117] |  | [0.623,1.190] |
|  |  |  |  |  |  |  |  |  |
| other accommodation | 0.971 | 0.973 |  | 1.162 |  | 1.019 |  | 0.951 |
|  | [0.670,1.409] | [0.671,1.412] |  | [0.814,1.659] |  | [0.787,1.321] |  | [0.650,1.392] |
|  |  |  |  |  |  |  |  |  |
| **Residence/asylum status** |  |  |  |  |  |  |  |  |
| r. no/unclear |  |  |  |  |  |  |  |  |
|  |  |  |  |  |  |  |  |  |
| other resid. permits | 0.965 | 0.949 |  | 0.791 |  | 1.136 |  | 1.128 |
|  | [0.444,2.099] | [0.436,2.065] |  | [0.412,1.519] |  | [0.709,1.820] |  | [0.501,2.538] |
|  |  |  |  |  |  |  |  |  |
| resid. permit (TPD) | 1.168 | 1.145 |  | 0.690 |  | 1.132 |  | 1.354 |
|  | [0.648,2.106] | [0.634,2.066] |  | [0.420,1.134] |  | [0.783,1.636] |  | [0.720,2.547] |
|  |  |  |  |  |  |  |  |  |
| **Partner's living place** |  |  |  |  |  |  |  |  |
| r. no partner |  |  |  |  |  |  |  |  |
|  |  |  |  |  |  |  |  |  |
| Germany | 1.008 | 1.013 |  | 1.076 |  | 1.056 |  | 1.027 |
|  | [0.807,1.260] | [0.811,1.266] |  | [0.873,1.325] |  | [0.915,1.218] |  | [0.818,1.289] |
|  |  |  |  |  |  |  |  |  |
| Ukraine/abroad | 0.805 | 0.811 |  | 0.749^*^ |  | 0.950 |  | 0.840 |
|  | [0.635,1.021] | [0.639,1.029] |  | [0.595,0.943] |  | [0.819,1.101] |  | [0.658,1.072] |
|  |  |  |  |  |  |  |  |  |
| **Children’s living place** |  |  |  |  |  |  |  |  |
| r. no child |  |  |  |  |  |  |  |  |
|  |  |  |  |  |  |  |  |  |
| one/all in Germany | 0.887 | 0.874 |  | 0.866 |  | 1.071 |  | 0.928 |
|  | [0.675,1.165] | [0.665,1.148] |  | [0.671,1.117] |  | [0.906,1.265] |  | [0.702,1.228] |
|  |  |  |  |  |  |  |  |  |
| one/all in Ukraine/abroad | 0.997 | 0.986 |  | 0.871 |  | 1.202 |  | 1.026 |
|  | [0.711,1.399] | [0.703,1.381] |  | [0.634,1.197] |  | [0.956,1.511] |  | [0.726,1.451] |
|  |  |  |  |  |  |  |  |  |
| **Perceived discrimination** |  |  |  |  |  |  |  |  |
| r. never |  |  |  |  |  |  |  |  |
|  |  |  |  |  |  |  |  |  |
| seldomly/often | **1.877^***^** | **1.877^***^** |  | **1.922^***^** |  | **1.572^***^** |  | **1.839^***^** |
|  | [1.557,2.262] | [1.557,2.262] |  | [1.610,2.294] |  | [1.398,1.768] |  | [1.520,2.225] |
|  |  |  |  |  |  |  |  |  |
| **Attended German language/integration course** |  |  |  |  |  |  |  |  |
| r. no |  |  |  |  |  |  |  |  |
|  |  |  |  |  |  |  |  |  |
| yes | **0.715^**^** | **0.715^**^** |  | **0.772^**^** |  | **0.842^*^** |  | **0.711^**^** |
|  | [0.585,0.875] | [0.585,0.875] |  | [0.635,0.940] |  | [0.732,0.969] |  | [0.579,0.872] |
|  |  |  |  |  |  |  |  |  |
| **German language proficiency** |  |  |  |  |  |  |  |  |
| r. none/poor |  |  |  |  |  |  |  |  |
|  |  |  |  |  |  |  |  |  |
| sufficient | **0.730^*^** | **0.728^*^** |  | **0.703^*^** |  | **0.594^***^** |  | **0.714^*^** |
|  | [0.553,0.964] | [0.552,0.962] |  | [0.537,0.919] |  | [0.512,0.689] |  | [0.539,0.946] |
|  |  |  |  |  |  |  |  |  |
| good/excellent | **0.375^**^** | **0.371^**^** |  | 0.688 |  | **0.534^***^** |  | **0.334^***^** |
|  | [0.205,0.685] | [0.203,0.678] |  | [0.440,1.076] |  | [0.420,0.679] |  | [0.179,0.626] |
|  |  |  |  |  |  |  |  |  |
| **Feeling socially isolated** |  |  |  |  |  |  |  |  |
| r. does not apply (at all)/neutral position |  |  |  |  |  |  |  |  |
|  |  |  |  |  |  |  |  |  |
| applies (fully) | **2.688^***^** | **2.691^***^** |  | **2.273^***^** |  | **2.037^***^** |  | **2.691^***^** |
|  | [2.226,3.248] | [2.228,3.251] |  | [1.896,2.726] |  | [1.783,2.329] |  | [2.219,3.264] |
|  |  |  |  |  |  |  |  |  |
| **Contact with Ukrainians(non-relative)** |  |  |  |  |  |  |  |  |
| r. no/seldomly |  |  |  |  |  |  |  |  |
|  |  |  |  |  |  |  |  |  |
| often | **0.728^*^** | **0.729^*^** |  | **0.743^*^** |  | 0.959 |  | **0.710^**^** |
|  | [0.563,0.941] | [0.564,0.943] |  | [0.581,0.950] |  | [0.806,1.141] |  | [0.547,0.921] |
|  |  |  |  |  |  |  |  |  |
| very often | **0.772^*^** | **0.774^*^** |  | 0.807 |  | **0.816^*^** |  | **0.777^*^** |
|  | [0.616,0.968] | [0.617,0.971] |  | [0.650,1.003] |  | [0.698,0.955] |  | [0.618,0.978] |
|  |  |  |  |  |  |  |  |  |
| **Contact with Germans** |  |  |  |  |  |  |  |  |
| r. no/seldomly |  |  |  |  |  |  |  |  |
|  |  |  |  |  |  |  |  |  |
| often | 0.929 | 0.926 |  | 1.065 |  | 0.902 |  | 0.915 |
|  | [0.738,1.169] | [0.736,1.166] |  | [0.855,1.327] |  | [0.776,1.048] |  | [0.724,1.156] |
|  |  |  |  |  |  |  |  |  |
| very often | **0.670^***^** | **0.670^***^** |  | **0.809^*^** |  | **0.755^***^** |  | **0.667^***^** |
|  | [0.538,0.835] | [0.539,0.835] |  | [0.658,0.994] |  | [0.660,0.863] |  | [0.534,0.833] |
|  |  |  |  |  |  |  |  |  |
| **Observations** | 5932 | 5932 |  | 5920 |  | 5932 |  | 5676 |
| **Pseudo *R*^2^** | - | - |  | - |  | - |  | 0.100 |
| **Average degrees of freedom** | 6957722.19 | 31400000 |  | 28800000 |  | 8828223.20 |  | - |
| **Model F-value** | 11.21 | 11.59 |  | 10.55 |  | 23.08 |  | - |
| **Model p-value (F-test)** | 0.0000 | 0.0000 |  | 0.0000 |  | 0.0000 |  | - |
| **Largest FMI** | 0.0492 | 0.0490 |  | 0.0408 |  | 0.0346 |  | - |
| Point estimates represent odds rations from multiple logistic regression models. Number in brackets represent 95% confidence intervals. Data are multiply imputed using 30 iterations.  ^*^ *p* < 0.05, ^**^ *p* < 0.01, ^***^ *p* < 0.001  Abbreviations: ACLED-Armed Conflict Location & Data Event Data; PTS-Political Terror Scale; TPD-Temporary Protection Directive; FMI-Fraction of Missing Information  ^a^ Self-rated health is binary coded so that value “*0*” (good/satisf) includes categories “*satisfactory*”, “*good*”, and “*very good*”, while value “*1*” (bad) includes categories “*bad*”, and “*very bad*” ^b^ Concerned of health situation is binary coded so that value “*0*” includes categories “*little concerned*” and “*not at all*”, while value “*1*” includes the only category “*very concerned*” ^c^ Self-rated health is binary coded so that value “*0*” (good) includes categories “*good*”, and “*very good*”, while value “*1*” (satisf/bad) includes categories “*satisfactory*”, “*bad*”, and “*very bad*” | | | | | | | | |

#

# Table G: Regression results sensitivity analyses (SA6) – Pre- and post-migration determinants of self-rated health with migration cohorts as an additional covariate, Ukrainian refugees

| Ukrainian refugees  (imputed data) | **General health**  **(self-rated)** | |
| --- | --- | --- |
|  | **SA6_Ukr_** |  |
|  | **Controlling for Migration cohorts** |  |
|  |  |  |
| SOCIODEMOGRAPHICS | | |
|  |  |  |
| **Gender** |  |  |
| r. male |  |  |
|  |  |  |
| female | 0.949 |  |
|  | [0.744,1.211] |  |
|  |  |  |
| **Age group** |  |  |
| r. 18-30 |  |  |
|  |  |  |
| 31-49 | 1.000 |  |
|  | [0.744,1.346] |  |
|  |  |  |
| 50+ | **2.068^***^** |  |
|  | [1.469,2.912] |  |
|  |  |  |
| **Economic situation before flight** |  |  |
| r. (well)below average |  |  |
|  |  |  |
| on average | **0.770^*^** |  |
|  | [0.615,0.963] |  |
|  |  |  |
| (well)above average | **0.632^***^** |  |
|  | [0.482,0.828] |  |
|  |  |  |
| **Level of education** |  |  |
| r. low |  |  |
|  |  |  |
| medium | 0.998 |  |
|  | [0.729,1.367] |  |
|  |  |  |
| high | 0.780 |  |
|  | [0.604,1.006] |  |
|  |  |  |
| **Employed before flight** |  |  |
| r. no |  |  |
|  |  |  |
| yes | **0.744^*^** |  |
|  | [0.591,0.937] |  |
|  |  |  |
| PRE-MIGRATION FACTORS | | |
|  |  |  |
| **PTS** |  |  |
| r. low (<=3) |  |  |
|  |  |  |
| high (>3) | 0.987 |  |
|  | [0.825,1.179] |  |
|  |  |  |
| **Traumatic experiences before flight** |  |  |
| r. none |  |  |
|  |  |  |
| one/more | 0.824 |  |
|  | [0.559,1.213] |  |
|  |  |  |
| POST-MIGRATION FACTORS | | |
|  |  |  |
| **Time in Germany since arrival** |  |  |
| r. 0-6 months |  |  |
|  |  |  |
| 6-12 months | 0.676 |  |
|  | [0.247,1.852] |  |
|  |  |  |
| **Type of accomodation** |  |  |
| r. shared accom. for refugees |  |  |
|  |  |  |
| private apartment/house | 0.860 |  |
|  | [0.626,1.181] |  |
|  |  |  |
| other accommodation | 0.973 |  |
|  | [0.671,1.412] |  |
|  |  |  |
| **Residence/asylum status** |  |  |
| r. no/unclear |  |  |
|  |  |  |
| other resid. permits | 0.929 |  |
|  | [0.427,2.020] |  |
|  |  |  |
| resid. Permit (TPD, §24 AsylbLG) | 1.163 |  |
|  | [0.645,2.094] |  |
|  |  |  |
| **Partner's living place** |  |  |
| r. no partner |  |  |
|  |  |  |
| Germany | 1.011 |  |
|  | [0.810,1.263] |  |
|  |  |  |
| Ukraine/abroad | 0.808 |  |
|  | [0.637,1.024] |  |
|  |  |  |
| **Children living place** |  |  |
| r. no child |  |  |
|  |  |  |
| one/all in Germany | 0.880 |  |
|  | [0.670,1.156] |  |
|  |  |  |
| one/all in Ukraine/abroad | 0.985 |  |
|  | [0.703,1.381] |  |
|  |  |  |
| **Perceived discrimination** |  |  |
| r. never |  |  |
|  |  |  |
| seldomly/often | **1.879^***^** |  |
|  | [1.559,2.264] |  |
|  |  |  |
| **Attended German language/integration course** |  |  |
| r. no |  |  |
|  |  |  |
| yes | **0.716^**^** |  |
|  | [0.585,0.877] |  |
|  |  |  |
| **German language proficiency** |  |  |
| r. none/poor |  |  |
|  |  |  |
| sufficient | **0.734^*^** |  |
|  | [0.556,0.969] |  |
|  |  |  |
| good/excellent | **0.374^**^** |  |
|  | [0.205,0.682] |  |
|  |  |  |
| **Feeling socially isolated** |  |  |
| r. does not apply (at all)/neutral pos. |  |  |
|  |  |  |
| applies (fully) | **2.696^***^** |  |
|  | [2.233,3.255] |  |
|  |  |  |
| **Contact with Ukrainians(non-relative)** |  |  |
| r. no/seldomly |  |  |
|  |  |  |
| often | **0.722^*^** |  |
|  | [0.559,0.934] |  |
|  |  |  |
| very often | **0.775^*^** |  |
|  | [0.618,0.971] |  |
|  |  |  |
| **Contact with Germans** |  |  |
| r. no/seldomly |  |  |
|  |  |  |
| often | 0.925 |  |
|  | [0.734,1.164] |  |
|  |  |  |
| very often | **0.668^***^** |  |
|  | [0.536,0.832] |  |
|  |  |  |
| **Migration cohorts** |  |  |
| r. arrived before 31May2022 |  |  |
|  |  |  |
| arrived after 31May2022 | 0.842 |  |
|  | [0.326,2.174] |  |
|  |  |  |
|  |  |  |
| **Observations** | 5932 |  |
| **Pseudo *R*^2^** | - |  |
| **Average degrees of freedom** | 64800000 |  |
| **Model F-value** | 11.93 |  |
| **Model p-value (F-test)** | 0.0000 |  |
| **Largest FMI** | 0.0440 |  |
| Odds ratios from multiple logistic regression models. Number in brackets represent 95% confidence intervals. Data are multiply imputed using 30 iterations. ^*^ *p* < 0.05, ^**^ *p* < 0.01, ^***^ *p* < 0.001; Abbreviations: PTS-Political Terror Scale; FMI-Fraction of Missing Information | |  |

# Table H: Regression results sensitivity analyses (SA4, SA5, SA7) – Pre- and post-migration determinants of self-rated health, non-Ukrainian refugees

|  | **General health**  **(self-rated)^a^** | |  | **General health**  **(self-rated)^b^** |
| --- | --- | --- | --- | --- |
|  | **SA4_non-Ukr_** | **SA7_Syr_** |  | **SA5_non-Ukr_** |
|  | fully adj. model (main model. All non-Ukrainian refugees) | fully adj. model (main model. Only Syrian refugees) |  | fully adj. model (main model. All non-Ukrainian refugees.  Data not imputed) |
|  |  |  |  |  |
| SOCIODEMOGRAPHICS | | | | |
|  |  |  |  |  |
| **Gender** |  |  |  |  |
| r. male |  |  |  |  |
|  |  |  |  |  |
| female | **1.730^**^** | 1.441 |  | **1.801^*^** |
|  | [1.226,2.441] | [0.907,2.290] |  | [1.128,2.877] |
|  |  |  |  |  |
| **Age group** |  |  |  |  |
| r. 18-30 |  |  |  |  |
|  |  |  |  |  |
| 31-49 | **2.183^***^** | **2.712^***^** |  | **2.217^**^** |
|  | [1.542,3.091] | [1.643,4.476] |  | [1.317,3.733] |
|  |  |  |  |  |
| 50+ | **6.696^***^** | **6.938^***^** |  | **4.374^***^** |
|  | [3.892,11.52] | [3.378,14.25] |  | [2.085,9.176] |
|  |  |  |  |  |
| **Economic situation before flight** |  |  |  |  |
| r. (well)below average |  |  |  |  |
|  |  |  |  |  |
| on average | **0.698^*^** | 0.723 |  | 0.688 |
|  | [0.493,0.989] | [0.447,1.169] |  | [0.421,1.123] |
|  |  |  |  |  |
| (well)above average | **0.559^**^** | **0.514^*^** |  | 0.942 |
|  | [0.369,0.845] | [0.292,0.906] |  | [0.542,1.636] |
|  |  |  |  |  |
| **Level of education** |  |  |  |  |
| r. low |  |  |  |  |
|  |  |  |  |  |
| medium | 0.778 | 0.967 |  | **0.538^*^** |
|  | [0.538,1.126] | [0.610,1.534] |  | [0.307,0.943] |
|  |  |  |  |  |
| high | 1.080 | 1.536 |  | 1.188 |
|  | [0.722,1.614] | [0.904,2.610] |  | [0.690,2.044] |
|  |  |  |  |  |
| **Employed before flight** |  |  |  |  |
| r. no |  |  |  |  |
|  |  |  |  |  |
| yes | 0.900 | 0.885 |  | 0.924 |
|  | [0.628,1.288] | [0.548,1.429] |  | [0.564,1.513] |
|  |  |  |  |  |
| **Country of birth** |  |  |  |  |
| r. Syria |  |  |  |  |
|  |  |  |  |  |
| Afghanistan | 1.055 |  |  | 0.505 |
|  | [0.638,1.746] |  |  | [0.189,1.348] |
|  |  |  |  |  |
| Iraq | 1.285 |  |  | 1.151 |
|  | [0.861,1.917] |  |  | [0.660,2.007] |
|  |  |  |  |  |
| Other countries | 0.700 |  |  | 0.897 |
|  | [0.446,1.098] |  |  | [0.485,1.660] |
|  |  |  |  |  |
| PRE-MIGRATION FACTORS | | | | |
|  |  |  |  |  |
| **PTS (avg)** |  |  |  |  |
| r. low (<=3) |  |  |  |  |
|  |  |  |  |  |
| high (>3) | 0.971 | 0.845 |  | 0.552 |
|  | [0.465,2.024] | [0.174,4.112] |  | [0.225,1.356] |
|  |  |  |  |  |
| **Reason for leaving the country** |  |  |  |  |
| r. other reasons |  |  |  |  |
|  |  |  |  |  |
| war, conflict and/or persecution | 0.813 | 0.762 |  | 0.696 |
|  | [0.573,1.154] | [0.473,1.228] |  | [0.416,1.164] |
|  |  |  |  |  |
| POST-MIGRATION FACTORS | | | | |
|  |  |  |  |  |
| **Time in Germany since arrival** |  |  |  |  |
| r. 0-6 months |  |  |  |  |
|  |  |  |  |  |
| 6-12 months | 1.201 | 1.621 |  | 1.560 |
|  | [0.686,2.103] | [0.763,3.441] |  | [0.612,3.976] |
|  |  |  |  |  |
| **Type of accommodation** |  |  |  |  |
| r. shared accom. for refugees |  |  |  |  |
|  |  |  |  |  |
| private apartment/house | **0.632^**^** | 0.682 |  | 0.681 |
|  | [0.459,0.870] | [0.453,1.028] |  | [0.427,1.086] |
|  |  |  |  |  |
| other accommodation | 1.919 | 2.356 |  | 1.368 |
|  | [0.480,7.678] | [0.326,17.05] |  | [0.283,6.624] |
|  |  |  |  |  |
| **Residence/asylum status** |  |  |  |  |
| r. no/awaiting outcome |  |  |  |  |
|  |  |  |  |  |
| other residence permit/'Duldung' | 1.287 | 1.404 |  | 1.104 |
|  | [0.825,2.009] | [0.746,2.643] |  | [0.589,2.069] |
|  |  |  |  |  |
| residence permit (temp./perm.) | 1.054 | 1.215 |  | 1.132 |
|  | [0.752,1.479] | [0.755,1.956] |  | [0.703,1.821] |
|  |  |  |  |  |
| **Partner's living place** |  |  |  |  |
| r. no partner |  |  |  |  |
|  |  |  |  |  |
| Germany | 0.960 | 0.828 |  | 0.737 |
|  | [0.610,1.512] | [0.439,1.560] |  | [0.401,1.354] |
|  |  |  |  |  |
| country of origin/abroad | 1.124 | 1.188 |  | 0.938 |
|  | [0.657,1.921] | [0.584,2.416] |  | [0.469,1.875] |
|  |  |  |  |  |
| **Children’s living place** |  |  |  |  |
| r. no child |  |  |  |  |
|  |  |  |  |  |
| one/all in Germany | 0.997 | 0.979 |  | 0.732 |
|  | [0.652,1.527] | [0.559,1.715] |  | [0.404,1.325] |
|  |  |  |  |  |
| one/all abroad/died | 1.197 | 1.085 |  | 0.937 |
|  | [0.698,2.053] | [0.540,2.181] |  | [0.458,1.914] |
|  |  |  |  |  |
| **Perceived discrimination** |  |  |  |  |
| r. never |  |  |  |  |
|  |  |  |  |  |
| seldomly/often | 1.324 | 1.475 |  | 1.034 |
|  | [0.975,1.799] | [0.972,2.240] |  | [0.676,1.581] |
|  |  |  |  |  |
| **Attended German language/integration course** |  |  |  |  |
| r. no |  |  |  |  |
|  |  |  |  |  |
| yes | 0.794 | 0.670 |  | 0.828 |
|  | [0.588,1.072] | [0.448,1.003] |  | [0.536,1.278] |
|  |  |  |  |  |
| **German language proficiency** |  |  |  |  |
| r. none/poor |  |  |  |  |
|  |  |  |  |  |
| sufficient | 0.978 | 0.825 |  | 1.011 |
|  | [0.681,1.405] | [0.510,1.334] |  | [0.603,1.693] |
|  |  |  |  |  |
| good/excellent | 0.688 | 0.778 |  | 1.048 |
|  | [0.331,1.431] | [0.328,1.844] |  | [0.409,2.687] |
|  |  |  |  |  |
| **Feeling socially isolated** |  |  |  |  |
| r. never/sometimes |  |  |  |  |
|  |  |  |  |  |
| (very)often | **1.889^***^** | **1.595^*^** |  | **1.991^**^** |
|  | [1.363,2.618] | [1.007,2.527] |  | [1.291,3.072] |
|  |  |  |  |  |
| **Contact with persons with same country of origin (non-relative)** |  |  |  |  |
| r. no/seldomly |  |  |  |  |
|  |  |  |  |  |
| often | 1.148 | 0.926 |  | 1.213 |
|  | [0.761,1.733] | [0.533,1.610] |  | [0.670,2.197] |
|  |  |  |  |  |
| very often | 1.182 | 1.141 |  | 0.993 |
|  | [0.843,1.659] | [0.718,1.812] |  | [0.613,1.609] |
|  |  |  |  |  |
| **Contact with Germans** |  |  |  |  |
| r. no/seldomly |  |  |  |  |
|  |  |  |  |  |
| often | 1.100 | 0.944 |  | 1.034 |
|  | [0.754,1.606] | [0.570,1.563] |  | [0.612,1.747] |
|  |  |  |  |  |
| very often | 0.948 | 0.888 |  | 0.887 |
|  | [0.679,1.323] | [0.565,1.395] |  | [0.545,1.443] |
|  |  |  |  |  |
| **Observations** | 1192 | 670 |  | 980 |
| **Pseudo *R*^2^** | - | - |  | 0.098 |
| **Average degrees of freedom** | 3181155.37 | 2835839.53 |  | - |
| **Model F-value** | 4.04 | 2.51 |  | - |
| **Model p-value (F-test)** | 0.0000 | 0.0000 |  | - |
| **Largest FMI** | 0.0658 | 0.0578 |  | - |

Point estimates represent odds rations from multiple logistic regression models. Number in brackets represent 95% confidence intervals. Data are multiply imputed using 30 iterations.

^*^ *p* < 0.05, ^**^ *p* < 0.01, ^***^ *p* < 0.001

Abbreviations: PTS-Political Terror Scale; FMI-Fraction of Missing Information

^a^ Self-rated health is binary coded so that value “*0*” (good) includes categories “*good*”, and “*very good*”, while value “*1*” (satisf/bad) includes categories “*satisfactory*”, “*bad*”, and “*very bad*”

^b^ Self-rated health is binary coded so that value “*0*” (good/satisf) includes categories “*satisfactory*”, “*good*”, and “*very good*”, while value “*1*” (bad) includes categories “*bad*”, and “*very bad*”

# Table J: Regression results sensitivity analyses (SA8)– Pre- and post-migration determinants of self-rated health stratified along level of educational, Ukrainian refugees

| Ukrainian refugees | **Self-rated health** | | |  |
| --- | --- | --- | --- | --- |
|  | **SA8_Ukr_low_** | **SA8_Ukr_med_** | **SA8_Ukr_high_** |  |
|  | **Level of education: Low** | **Level of education: Medium^a^** | **Level of education: High** |  |
|  |  |  |  |  |
| SOCIODEMOGRAPHICS | | | | |
|  |  |  |  |  |
| **Gender** |  |  |  |  |
| r. male |  |  |  |  |
|  |  |  |  |  |
| female | 1.677 | 0.966 | 0.835 |  |
|  | [0.869,3.234] | [0.542,1.724] | [0.616,1.133] |  |
|  |  |  |  |  |
| **Age group** |  |  |  |  |
| r. 18-30 |  |  |  |  |
|  |  |  |  |  |
| 31-49 | 1.294 | 1.797 | 0.852 |  |
|  | [0.608,2.755] | [0.760,4.250] | [0.593,1.224] |  |
|  |  |  |  |  |
| 50+ | 2.013 | 2.102 | **2.204^***^** |  |
|  | [0.806,5.030] | [0.860,5.133] | [1.444,3.364] |  |
|  |  |  |  |  |
| **Economic situation before flight** |  |  |  |  |
| r. (well)below average |  |  |  |  |
|  |  |  |  |  |
| on average | 1.049 | 0.637 | **0.675^**^** |  |
|  | [0.616,1.787] | [0.391,1.038] | [0.503,0.908] |  |
|  |  |  |  |  |
| (well)above average | 0.977 | **0.327^**^** | **0.595^**^** |  |
|  | [0.493,1.938] | [0.148,0.723] | [0.425,0.831] |  |
|  |  |  |  |  |
| **Employed before flight** |  |  |  |  |
| r. no |  |  |  |  |
|  |  |  |  |  |
| yes | **0.464^**^** | 0.650 | 0.955 |  |
|  | [0.285,0.755] | [0.367,1.151] | [0.688,1.325] |  |
|  |  |  |  |  |
| PRE-MIGRATION FACTORS | | | | |
|  |  |  |  |  |
| **PTS** |  |  |  |  |
| r. low (<=3) |  |  |  |  |
|  |  |  |  |  |
| high (>3) | 0.966 | 1.092 | 0.960 |  |
|  | [0.617,1.510] | [0.691,1.727] | [0.771,1.195] |  |
|  |  |  |  |  |
| **Traumatic experiences before flight** |  |  |  |  |
| r. none |  |  |  |  |
|  |  |  |  |  |
| one/more | 1.394 | 0.829 | 0.716 |  |
|  | [0.494,3.933] | [0.373,1.843] | [0.431,1.188] |  |
|  |  |  |  |  |
| POST-MIGRATION FACTORS | | | | |
|  |  |  |  |  |
| **Time in Germany since arrival** |  |  |  |  |
| r. 0-6 months |  |  |  |  |
|  |  |  |  |  |
| 6-12 months | 0.628 | 0.684 | 0.913 |  |
|  | [0.293,1.344] | [0.292,1.607] | [0.553,1.509] |  |
|  |  |  |  |  |
| **Type of accomodation** |  |  |  |  |
| r. shared accom. for refugees |  |  |  |  |
|  |  |  |  |  |
| private apartment/house | 1.224 | 0.656 | 0.869 |  |
|  | [0.526,2.850] | [0.332,1.299] | [0.576,1.309] |  |
|  |  |  |  |  |
| other accommodation | 2.317 | 0.673 | 0.884 |  |
|  | [0.896,5.992] | [0.285,1.587] | [0.548,1.427] |  |
|  |  |  |  |  |
| **Residence/asylum status** |  |  |  |  |
| r. no/unclear |  |  |  |  |
|  |  |  |  |  |
| other resid. permits | 2.311 | 0.185 | 1.375 |  |
|  | [0.480,11.11] | [0.0264,1.298] | [0.399,4.736] |  |
|  |  |  |  |  |
| resid. Permit (TPD, §24 AsylbLG) | 1.521 | 0.474 | 2.064 |  |
|  | [0.486,4.764] | [0.156,1.438] | [0.723,5.893] |  |
|  |  |  |  |  |
| **Partner's living place** |  |  |  |  |
| r. no partner |  |  |  |  |
|  |  |  |  |  |
| Germany | 0.946 | 0.984 | 1.046 |  |
|  | [0.537,1.665] | [0.565,1.711] | [0.792,1.382] |  |
|  |  |  |  |  |
| Ukraine/abroad | 1.009 | **0.469^*^** | 0.889 |  |
|  | [0.562,1.811] | [0.249,0.885] | [0.664,1.191] |  |
|  |  |  |  |  |
| **Children living place** |  |  |  |  |
| r. no child |  |  |  |  |
|  |  |  |  |  |
| one/all in Germany | 0.989 | 0.701 | 0.924 |  |
|  | [0.471,2.081] | [0.334,1.472] | [0.664,1.286] |  |
|  |  |  |  |  |
| one/all in Ukraine/abroad | 1.187 | 1.237 | 0.879 |  |
|  | [0.470,3.001] | [0.543,2.818] | [0.575,1.344] |  |
|  |  |  |  |  |
| **Perceived discrimination** |  |  |  |  |
| r. never |  |  |  |  |
|  |  |  |  |  |
| seldomly/often | **2.266^***^** | **1.726^*^** | **1.848^***^** |  |
|  | [1.425,3.604] | [1.051,2.836] | [1.468,2.327] |  |
|  |  |  |  |  |
| **Attended German language/integration course** |  |  |  |  |
| r. no |  |  |  |  |
|  |  |  |  |  |
| yes | 0.954 | **0.568^*^** | **0.667^**^** |  |
|  | [0.577,1.576] | [0.340,0.950] | [0.517,0.860] |  |
|  |  |  |  |  |
| **German language proficiency** |  |  |  |  |
| r. none/poor |  |  |  |  |
|  |  |  |  |  |
| sufficient | 0.707 |  | 0.746 |  |
|  | [0.372,1.342] |  | [0.533,1.043] |  |
|  |  |  |  |  |
| good/excellent | 0.310 |  | **0.403^**^** |  |
|  | [0.0852,1.125] |  | [0.202,0.803] |  |
|  |  |  |  |  |
| **Feeling socially isolated** |  |  |  |  |
| r. does not apply (at all)/neutral pos. |  |  |  |  |
|  |  |  |  |  |
| applies (fully) | **3.032^***^** | **2.698^***^** | **2.714^***^** |  |
|  | [1.928,4.768] | [1.635,4.453] | [2.149,3.428] |  |
|  |  |  |  |  |
| **Contact with Ukrainians(non-relative)** |  |  |  |  |
| r. no/seldomly |  |  |  |  |
|  |  |  |  |  |
| often | 0.604 | 0.509 | 0.848 |  |
|  | [0.323,1.132] | [0.259,1.002] | [0.617,1.166] |  |
|  |  |  |  |  |
| very often | **0.480^**^** | 0.603 | 0.982 |  |
|  | [0.278,0.828] | [0.339,1.073] | [0.737,1.308] |  |
|  |  |  |  |  |
| **Contact with Germans** |  |  |  |  |
| r. no/seldomly |  |  |  |  |
|  |  |  |  |  |
| often | 1.129 | 1.045 | 0.844 |  |
|  | [0.614,2.078] | [0.571,1.911] | [0.638,1.116] |  |
|  |  |  |  |  |
| very often | 1.068 | 0.636 | **0.575^***^** |  |
|  | [0.624,1.828] | [0.357,1.132] | [0.438,0.755] |  |
|  |  |  |  |  |
| **Observations** | 824 | 751 | 4348 |  |
| **Pseudo *R*^2^** | - | - | - |  |
| **Average degrees of freedom** | 7433720.61 | 7953619.51 | 18000000 |  |
| **Model F-value** | 2.68 | 2.96 | 8.47 |  |
| **Model p-value (F-test)** | 0.0000 | 0.0000 | 0.0000 |  |
| **Largest FMI** | 0.0313 | 0.0413 | 0.0314 |  |
| Point estimates represent odds rations from multiple logistic regression models. Number in brackets represent 95% confidence intervals. Data are multiply imputed using 30 iterations.  ^*^ *p* < 0.05, ^**^ *p* < 0.01, ^***^ *p* < 0.001  Abbreviations: PTS-Political Terror Scale; FMI-Fraction of Missing Information  ^a^ Language proficiency could not be considered as a covariate of this model due to low case numbers: nobody with a bad health reported good/excellent proficiency. | | | |  |

# Table J: Regression results sensitivity analyses (SA8)– Pre- and post-migration determinants of self-rated health stratified along level of educational, non-Ukrainian refugees

| Non-Ukrainian refugees | **Self-rated health** | | |  |
| --- | --- | --- | --- | --- |
|  | **SA8_non-Ukr_low_** | **SA8_non-Ukr_med_** | **SA8_non-Ukr_high_** |  |
|  | **Level of education: Low** | **Level of education: Medium** | **Level of education: High** |  |
|  |  |  |  |  |
| SOCIODEMOGRAPHICS | | | | |
|  |  |  |  |  |
| **Gender** |  |  |  |  |
| r. male |  |  |  |  |
|  |  |  |  |  |
| female | 1.725 | 3.151 | 1.403 |  |
|  | [0.932,3.193] | [0.927,10.71] | [0.521,3.778] |  |
|  |  |  |  |  |
| **Age group** |  |  |  |  |
| r. 18-30 |  |  |  |  |
|  |  |  |  |  |
| 31-49 | **2.210^**^** | 1.148 | **4.076^*^** |  |
|  | [1.247,3.916] | [0.279,4.722] | [1.028,16.16] |  |
|  |  |  |  |  |
| 50+ | **4.298^***^** | 4.153 | 4.544 |  |
|  | [1.891,9.769] | [0.600,28.74] | [0.371,55.71] |  |
|  |  |  |  |  |
| **Economic situation before flight** |  |  |  |  |
| r. (well)below average |  |  |  |  |
|  |  |  |  |  |
| on average | 0.825 | 0.583 | 0.764 |  |
|  | [0.476,1.430] | [0.151,2.249] | [0.186,3.139] |  |
|  |  |  |  |  |
| (well)above average | 0.969 | 0.824 | 0.668 |  |
|  | [0.486,1.933] | [0.199,3.413] | [0.171,2.601] |  |
|  |  |  |  |  |
| **Employed before flight** |  |  |  |  |
| r. no |  |  |  |  |
|  |  |  |  |  |
| yes | 0.732 | 1.220 | 1.187 |  |
|  | [0.391,1.370] | [0.338,4.401] | [0.343,4.114] |  |
|  |  |  |  |  |
| **Country of birth** |  |  |  |  |
| r. Syria |  |  |  |  |
|  |  |  |  |  |
| Afghanistan | 0.886 | 1.843 | 0.501 |  |
|  | [0.397,1.974] | [0.151,22.56] | [0.0387,6.471] |  |
|  |  |  |  |  |
| Iraq | 1.589 | 0.646 | 1.628 |  |
|  | [0.874,2.889] | [0.0585,7.123] | [0.322,8.235] |  |
|  |  |  |  |  |
| Other countries | 1.330 | 0.620 | 0.880 |  |
|  | [0.632,2.798] | [0.128,3.009] | [0.189,4.098] |  |
|  |  |  |  |  |
| PRE-MIGRATION FACTORS | | | | |
|  |  |  |  |  |
| **PTS (avg)** |  |  |  |  |
| r. low (<=3) |  |  |  |  |
|  |  |  |  |  |
| high (>3) | 0.792 | 0.181 | 0.345 |  |
|  | [0.251,2.501] | [0.0193,1.697] | [0.0486,2.445] |  |
|  |  |  |  |  |
| **Traumatic experiences before flight** |  |  |  |  |
| r. no |  |  |  |  |
|  |  |  |  |  |
| one/more | 0.645 | 0.599 | 0.653 |  |
|  | [0.351,1.185] | [0.150,2.381] | [0.193,2.200] |  |
|  |  |  |  |  |
| POST-MIGRATION FACTORS | | | | |
|  |  |  |  |  |
| **Time in Germany since arrival** |  |  |  |  |
| r. 0-6 months |  |  |  |  |
|  |  |  |  |  |
| 6-12 months | 0.884 | 1.427 | 1 |  |
|  | [0.377,2.073] | [0.0916,22.21] | [.,.] |  |
|  |  |  |  |  |
| **Type of accommodation** |  |  |  |  |
| r. shared accom. for refugees |  |  |  |  |
|  |  |  |  |  |
| private apartment/house | **0.490^*^** | 0.583 | 2.705 |  |
|  | [0.282,0.850] | [0.158,2.147] | [0.936,7.815] |  |
|  |  |  |  |  |
| other accommodation | 0.395 | 3.594 | 5.899 |  |
|  | [0.0272,5.747] | [0.0994,130.0] | [0.259,134.5] |  |
|  |  |  |  |  |
| **Residence/asylum status** |  |  |  |  |
| r. no/awaiting outcome |  |  |  |  |
|  |  |  |  |  |
| other residence permit/'Duldung' | 0.951 | 1.179 | 1.176 |  |
|  | [0.478,1.889] | [0.230,6.038] | [0.128,10.81] |  |
|  |  |  |  |  |
| residence permit (temp./perm.) | 0.782 | 1.017 | 2.316 |  |
|  | [0.450,1.361] | [0.264,3.917] | [0.751,7.141] |  |
|  |  |  |  |  |
| **Partner's living place** |  |  |  |  |
| r. no partner |  |  |  |  |
|  |  |  |  |  |
| Germany | 0.907 | 0.596 | 0.575 |  |
|  | [0.441,1.868] | [0.132,2.699] | [0.114,2.891] |  |
|  |  |  |  |  |
| country of origin/abroad | 1.373 | 1.188 | 0.487 |  |
|  | [0.587,3.208] | [0.237,5.963] | [0.0697,3.401] |  |
|  |  |  |  |  |
| **Children living place** |  |  |  |  |
| r. no child |  |  |  |  |
|  |  |  |  |  |
| one/all in Germany | 0.918 | 0.779 | 0.743 |  |
|  | [0.461,1.826] | [0.151,4.027] | [0.184,2.994] |  |
|  |  |  |  |  |
| one/all abroad/died | 1.189 | 1.332 | 0.770 |  |
|  | [0.530,2.666] | [0.210,8.430] | [0.0784,7.561] |  |
|  |  |  |  |  |
| **Perceived discrimination** |  |  |  |  |
| r. never |  |  |  |  |
|  |  |  |  |  |
| seldomly/often | 1.069 | 1.713 | 0.607 |  |
|  | [0.638,1.788] | [0.573,5.123] | [0.209,1.761] |  |
|  |  |  |  |  |
| **Attended German language/integration/ESF-BAMF course** |  |  |  |  |
| r. no |  |  |  |  |
|  |  |  |  |  |
| yes | 1.089 | 0.737 | **0.275^*^** |  |
|  | [0.663,1.789] | [0.236,2.300] | [0.0907,0.834] |  |
|  |  |  |  |  |
| **German language proficiency** |  |  |  |  |
| r. none/poor |  |  |  |  |
|  |  |  |  |  |
| sufficient | 1.028 | 1.759 | 0.496 |  |
|  | [0.512,2.064] | [0.530,5.838] | [0.164,1.503] |  |
|  |  |  |  |  |
| good/excellent | 0.751 | 2.645 | 0.939 |  |
|  | [0.161,3.508] | [0.226,31.02] | [0.192,4.579] |  |
|  |  |  |  |  |
| **Feeling socially isolated** |  |  |  |  |
| r. never/sometimes |  |  |  |  |
|  |  |  |  |  |
| (very)often | **2.232^**^** | 1.540 | **3.198^*^** |  |
|  | [1.347,3.698] | [0.466,5.096] | [1.018,10.04] |  |
|  |  |  |  |  |
| **Contact with persons with same country of origin (non-relative)** |  |  |  |  |
| r. no/seldomly |  |  |  |  |
|  |  |  |  |  |
| often | 1.028 | 2.733 | 0.603 |  |
|  | [0.513,2.062] | [0.567,13.18] | [0.148,2.464] |  |
|  |  |  |  |  |
| very often | 0.777 | 1.835 | 1.551 |  |
|  | [0.454,1.328] | [0.462,7.295] | [0.449,5.364] |  |
|  |  |  |  |  |
| **Contact with Germans** |  |  |  |  |
| r. no/seldomly |  |  |  |  |
|  |  |  |  |  |
| often | 1.015 | 0.559 | 1.194 |  |
|  | [0.545,1.889] | [0.150,2.087] | [0.304,4.683] |  |
|  |  |  |  |  |
| very often | 0.810 | 0.271 | 2.764 |  |
|  | [0.451,1.453] | [0.0645,1.138] | [0.845,9.033] |  |
|  |  |  |  |  |
| **Observations** | 720 | 253 | 207 |  |
| **Pseudo *R*^2^** | - | - | - |  |
| **Average degrees of freedom** | 3773514.71 | 2311018.83 | 435,445.53 |  |
| **Model F-value** | 1.98 | 0.71 | 0.79 |  |
| **Model p-value (F-test)** | 0.0013 | 0.8777 | 0.7712 |  |
| **Largest FMI** | 0.0749 | 0.2701 | 0.0730 |  |
| Point estimates represent odds rations from multiple logistic regression models. Number in brackets represent 95% confidence intervals. Data are multiply imputed using 30 iterations.  ^*^ *p* < 0.05, ^**^ *p* < 0.01, ^***^ *p* < 0.001  Abbreviations: PTS-Political Terror Scale; FMI-Fraction of Missing Information | | | |  |

# Table K: Regression results sensitivity analyses (SA9) – Pre- and post-migration determinants of self-rated health with interaction effects between gender and perceived discrimination, gender and social isolation, between age group and perceived discrimination, and age group and social isolation, Ukrainian refugees

| Ukrainian refugees (imputed data) | **Self-rated health** | |
| --- | --- | --- |
|  | **SA9_Ukr_** |  |
|  | **with interaction effects^a^** |  |
|  |  |  |
| SOCIODEMOGRAPHICS | | |
|  |  |  |
| **Gender** |  |  |
| r. male |  |  |
|  |  |  |
| female | 0.864 |  |
|  | [0.617,1.211] |  |
|  |  |  |
| **Age group** |  |  |
| r. 18-30 |  |  |
|  |  |  |
| 31-49 | 0.964 |  |
|  | [0.586,1.587] |  |
|  |  |  |
| 50+ | **2.546^***^** |  |
|  | [1.550,4.183] |  |
|  |  |  |
| **Economic situation before flight** |  |  |
| r. (well)below average |  |  |
|  |  |  |
| on average | **0.760^*^** |  |
|  | [0.608,0.951] |  |
|  |  |  |
| (well)above average | **0.625^***^** |  |
|  | [0.477,0.819] |  |
|  |  |  |
| **Level of education** |  |  |
| r. low |  |  |
|  |  |  |
| medium | 0.997 |  |
|  | [0.728,1.366] |  |
|  |  |  |
| high | **0.771^*^** |  |
|  | [0.597,0.995] |  |
|  |  |  |
| **Employed before flight** |  |  |
| r. no |  |  |
|  |  |  |
| yes | **0.751^*^** |  |
|  | [0.597,0.946] |  |
|  |  |  |
| PRE-MIGRATION FACTORS | | |
|  |  |  |
| **PTS** |  |  |
| r. low (<=3) |  |  |
|  |  |  |
| high (>3) | 0.994 |  |
|  | [0.831,1.187] |  |
|  |  |  |
| **Traumatic experiences before flight** |  |  |
| r. none |  |  |
|  |  |  |
| one/more | 0.821 |  |
|  | [0.557,1.210] |  |
|  |  |  |
| POST-MIGRATION FACTORS | | |
|  |  |  |
| **Time in Germany since arrival** |  |  |
| r. 0-6 months |  |  |
|  |  |  |
| 6-12 months | 0.796 |  |
|  | [0.552,1.148] |  |
|  |  |  |
| **Type of accommodation** |  |  |
| r. shared accom. for refugees |  |  |
|  |  |  |
| private apartment/house | 0.856 |  |
|  | [0.623,1.176] |  |
|  |  |  |
| other accommodation | 0.966 |  |
|  | [0.666,1.401] |  |
|  |  |  |
| **Residence/asylum status** |  |  |
| r. no/unclear |  |  |
|  |  |  |
| other resid. permits | 0.925 |  |
|  | [0.425,2.015] |  |
|  |  |  |
| resid. Permit (TPD, §24 AsylbLG) | 1.177 |  |
|  | [0.652,2.126] |  |
|  |  |  |
| **Partner's living place** |  |  |
| r. no partner |  |  |
|  |  |  |
| Germany | 1.003 |  |
|  | [0.803,1.254] |  |
|  |  |  |
| Ukraine/abroad | 0.809 |  |
|  | [0.638,1.026] |  |
|  |  |  |
| **Children living place** |  |  |
| r. no child |  |  |
|  |  |  |
| one/all in Germany | 0.884 |  |
|  | [0.672,1.161] |  |
|  |  |  |
| one/all in Ukraine/abroad | 0.996 |  |
|  | [0.711,1.396] |  |
|  |  |  |
| **Perceived discrimination** |  |  |
| r. never |  |  |
|  |  |  |
| seldomly/often | 1.194 |  |
|  | [0.679,2.097] |  |
|  |  |  |
| **Attended German language/integration course** |  |  |
| r. no |  |  |
|  |  |  |
| yes | **0.724^**^** |  |
|  | [0.591,0.886] |  |
|  |  |  |
| **German language proficiency** |  |  |
| r. none/poor |  |  |
|  |  |  |
| sufficient | **0.735^*^** |  |
|  | [0.556,0.973] |  |
|  |  |  |
| good/excellent | **0.370^**^** |  |
|  | [0.202,0.676] |  |
|  |  |  |
| **Feeling socially isolated** |  |  |
| r. does not apply (at all)/neutral pos. |  |  |
|  |  |  |
| applies (fully) | **4.206^***^** |  |
|  | [2.394,7.391] |  |
|  |  |  |
| **Contact with Ukrainians(non-relative)** |  |  |
| r. no/seldomly |  |  |
|  |  |  |
| often | **0.711^**^** |  |
|  | [0.551,0.919] |  |
|  |  |  |
| very often | **0.765^*^** |  |
|  | [0.610,0.960] |  |
|  |  |  |
| **Contact with Germans** |  |  |
| r. no/seldomly |  |  |
|  |  |  |
| often | 0.921 |  |
|  | [0.731,1.160] |  |
|  |  |  |
| very often | **0.664^***^** |  |
|  | [0.534,0.827] |  |
|  |  |  |
| INTERACTION EFFECTS | | |
|  |  |  |
| **Gender * Feeling socially isolated** |  |  |
| r. male # does not apply (at all)/neutral pos. |  |  |
|  |  |  |
| female # applies (fully) | 0.735 |  |
|  | [0.451,1.198] |  |
|  |  |  |
| **Gender * Perceived discrimination** |  |  |
| r. male # never |  |  |
|  |  |  |
| female # seldomly/often | **1.645^*^** |  |
|  | [1.020,2.652] |  |
|  |  |  |
| **Age group * Feeling socially isolated** |  |  |
| r. 10-30 # does not apply (at all)/neutral pos. |  |  |
|  |  |  |
| 31-49 # applies (fully) | 0.934 |  |
|  | [0.584,1.492] |  |
|  |  |  |
| 50+ # applies (fully) | **0.597^*^** |  |
|  | [0.359,0.993] |  |
|  |  |  |
| **Age group * Perceived discrimination** |  |  |
| r. 18-30 # never |  |  |
|  |  |  |
| 31-49 # seldomly/often | 1.146 |  |
|  | [0.706,1.860] |  |
|  |  |  |
| 50+ # seldomly/often | 0.948 |  |
|  | [0.567,1.587] |  |
|  |  |  |
| **Observations** | 5932 |  |
| **Pseudo *R*^2^** | - |  |
| **Average degrees of freedom** | 12800000 |  |
| **Model F-value** | 10.19 |  |
| **Model p-value (F-test)** | 0.0000 |  |
| **Largest FMI** | 0.0354 |  |
| Point estimates represent odds rations from multiple logistic regression models. Number in brackets represent 95% confidence intervals. Data are multiply imputed using 30 iterations.  ^*^ *p* < 0.05, ^**^ *p* < 0.01, ^***^ *p* < 0.001  Abbreviations: PTS-Political Terror Scale; FMI-Fraction of Missing Information  ^a^ Interaction terms between gender and language proficiency as well as between age group and language proficiency could not be considered due to low case numbers: only two males with bad health reported good/excellent proficiency and only ten females, along age groups very few persons with bad health reported good/excellent proficiency: six in the age group 18-30, six in the age group 31-49, nobody in the age group 50+. | |  |

# Table L: Regression results sensitivity analyses (SA9) – Pre- and post-migration determinants of self-rated health with interaction effects between gender and perceived discrimination, gender and social isolation, between age group and perceived discrimination, and age group and social isolation, non-Ukrainian refugees

|  | **Self-rated health** | |
| --- | --- | --- |
|  | **SA9_non-Ukr_** |  |
|  | **with interaction effects** |  |
|  |  |  |
| SOCIODEMOGRAPHICS | | |
|  |  |  |
| **Gender** |  |  |
| r. male |  |  |
|  |  |  |
| female | **2.334^**^** |  |
|  | [1.240,4.393] |  |
|  |  |  |
| **Age group** |  |  |
| r. 18-30 |  |  |
|  |  |  |
| 31-49 | **1.983^*^** |  |
|  | [1.040,3.780] |  |
|  |  |  |
| 50+ | **5.052^***^** |  |
|  | [2.101,12.15] |  |
|  |  |  |
| **Economic situation before flight** |  |  |
| r. (well)below average |  |  |
|  |  |  |
| on average | 0.700 |  |
|  | [0.441,1.111] |  |
|  |  |  |
| (well)above average | 0.846 |  |
|  | [0.499,1.436] |  |
|  |  |  |
| **Level of education** |  |  |
| r. low |  |  |
|  |  |  |
| medium | 0.623 |  |
|  | [0.366,1.062] |  |
|  |  |  |
| high | 1.216 |  |
|  | [0.727,2.035] |  |
|  |  |  |
| **Employed before flight** |  |  |
| r. no |  |  |
|  |  |  |
| yes | 0.991 |  |
|  | [0.620,1.582] |  |
|  |  |  |
| **Country of birth** |  |  |
| r. Syria |  |  |
|  |  |  |
| Afghanistan | 0.863 |  |
|  | [0.424,1.760] |  |
|  |  |  |
| Iraq | 1.429 |  |
|  | [0.853,2.394] |  |
|  |  |  |
| Other countries | 1.004 |  |
|  | [0.569,1.774] |  |
|  |  |  |
| PRE-MIGRATION FACTORS | | |
|  |  |  |
| **PTS (avg)** |  |  |
| r. low (<=3) |  |  |
|  |  |  |
| high (>3) | 0.498 |  |
|  | [0.214,1.158] |  |
|  |  |  |
| **Traumatic experiences before flight** |  |  |
| r. no |  |  |
|  |  |  |
| one/more | 0.698 |  |
|  | [0.430,1.134] |  |
|  |  |  |
| POST-MIGRATION FACTORS | | |
|  |  |  |
| **Time in Germany since arrival** |  |  |
| r. 0-6 months |  |  |
|  |  |  |
| 6-12 months | 1.165 |  |
|  | [0.529,2.566] |  |
|  |  |  |
| **Type of accommodation** |  |  |
| r. shared accom. for refugees |  |  |
|  |  |  |
| private apartment/house | **0.648^*^** |  |
|  | [0.422,0.997] |  |
|  |  |  |
| other accommodation | 1.344 |  |
|  | [0.255,7.071] |  |
|  |  |  |
| **Residence/asylum status** |  |  |
| r. no/awaiting outcome |  |  |
|  |  |  |
| other residence permit/'Duldung' | 1.034 |  |
|  | [0.576,1.854] |  |
|  |  |  |
| residence permit (temp./perm.) | 1.045 |  |
|  | [0.671,1.628] |  |
|  |  |  |
| **Partner's living place** |  |  |
| r. no partner |  |  |
|  |  |  |
| Germany | 0.695 |  |
|  | [0.387,1.248] |  |
|  |  |  |
| country of origin/abroad | 1.138 |  |
|  | [0.583,2.221] |  |
|  |  |  |
| **Children living place** |  |  |
| r. no child |  |  |
|  |  |  |
| one/all in Germany | 0.898 |  |
|  | [0.514,1.569] |  |
|  |  |  |
| one/all abroad/died | 1.027 |  |
|  | [0.520,2.027] |  |
|  |  |  |
| **Perceived discrimination** |  |  |
| r. never |  |  |
|  |  |  |
| seldomly/often | 0.775 |  |
|  | [0.372,1.617] |  |
|  |  |  |
| **Attended German language/integration/ESF-BAMF course** |  |  |
| r. no |  |  |
|  |  |  |
| yes | 0.779 |  |
|  | [0.521,1.166] |  |
|  |  |  |
| **German language proficiency** |  |  |
| r. none/poor |  |  |
|  |  |  |
| sufficient | 1.519 |  |
|  | [0.689,3.346] |  |
|  |  |  |
| good/excellent | 0.754 |  |
|  | [0.160,3.551] |  |
|  |  |  |
| **Feeling socially isolated** |  |  |
| r. never/sometimes |  |  |
|  |  |  |
| (very)often | **2.606^*^** |  |
|  | [1.237,5.494] |  |
|  |  |  |
| **Contact with persons with same country of origin (non-relative)** |  |  |
| r. no/seldomly |  |  |
|  |  |  |
| often | 1.203 |  |
|  | [0.698,2.073] |  |
|  |  |  |
| very often | 1.087 |  |
|  | [0.693,1.704] |  |
|  |  |  |
| **Contact with Germans** |  |  |
| r. no/seldomly |  |  |
|  |  |  |
| often | 0.900 |  |
|  | [0.544,1.487] |  |
|  |  |  |
| very often | 0.951 |  |
|  | [0.607,1.490] |  |
|  |  |  |
| INTERACTION EFFECTS | | |
|  |  |  |
| **Gender * Feeling socially isolated** |  |  |
| r. male # never/sometimes |  |  |
|  |  |  |
| female # (very)often | 0.593 |  |
|  | [0.259,1.358] |  |
|  |  |  |
| **Gender * Perceived discrimination** |  |  |
| r. male # never |  |  |
|  |  |  |
| female # seldomly/often | 2.156 |  |
|  | [0.955,4.868] |  |
|  |  |  |
| **Gender * German language proficiency** |  |  |
| r. male # none/poor |  |  |
|  |  |  |
| female # sufficient | 0.563 |  |
|  | [0.215,1.474] |  |
|  |  |  |
| female # good/excellent | 0.111 |  |
|  | [0.00998,1.236] |  |
|  |  |  |
| **Age group * Feeling socially isolated** |  |  |
| r. 10-30 # never/sometimes |  |  |
|  |  |  |
| 31-49 # (very)often | 1.064 |  |
|  | [0.435,2.606] |  |
|  |  |  |
| 50+ # (very)often | 1.965 |  |
|  | [0.552,7.001] |  |
|  |  |  |
| **Age group * Perceived discrimination** |  |  |
| r. 18-30 # never |  |  |
|  |  |  |
| 31-49 # seldomly/often | 0.795 |  |
|  | [0.333,1.901] |  |
|  |  |  |
| 50+ # seldomly/often | 0.595 |  |
|  | [0.155,2.283] |  |
|  |  |  |
| **Age group * German language proficiency** |  |  |
| r. 18-30 # none/poor |  |  |
|  |  |  |
| 31-49 # sufficient | 1.001 |  |
|  | [0.383,2.619] |  |
|  |  |  |
| 31-49 # good/excellent | **8.526^*^** |  |
|  | [1.204,60.40] |  |
|  |  |  |
| 50+ # sufficient | *omitted* |  |
|  |  |  |
|  |  |  |
| 50+ # good/excellent | *omitted* |  |
|  |  |  |
|  |  |  |
| **Observations** | 1176 |  |
| **Pseudo *R*^2^** | - |  |
| **Average degrees of freedom** | 5487911.95 |  |
| **Model F-value** | 2.18 |  |
| **Model p-value (F-test)** | 0.0000 |  |
| **Largest FMI** | 0.0843 |  |
| Point estimates represent odds rations from multiple logistic regression models. Number in brackets represent 95% confidence intervals. Data are multiply imputed using 30 iterations.  ^*^ *p* < 0.05, ^**^ *p* < 0.01, ^***^ *p* < 0.001  Abbreviations: PTS-Political Terror Scale; FMI-Fraction of Missing Information | |  |
