# Supplementary material for: Pre- and post-migration determinants of self-rated health among Ukrainian refugees in Germany: A cross-sectional comparative analysis with recently arrived refugees from other countries of origin
Source: PLOS Glob Public Health. 2025 Nov 10;5(11):e0004565. doi: 10.1371/journal.pgph.0004565 (PMC12599935; doi:10.1371/journal.pgph.0004565)
Supplement: Attachment — Submitted filename: Request_Edits.docx [file pgph.0004565.s002.docx]

| 1. This appears to be a resubmission from PLOS Medicine. Please include a rebuttal letter with a response to the decision and reviewers’ comments (if applicable), detailing any revisions made to the original submission. Please upload this as a 'Response to Reviewers' file type. | We had previously submitted this Manuscript to PLOS Medicine but it was rejected prior to per review. We therefore made no alterations to the manuscript |
| --- | --- |
| 2. Please complete all items on the Human Participants Research Checklist that are relevant for your submission, by following this link: <http://journals.plos.org/plosone/s/file?id=dc11/PLOSOne_Human_Subjects_Research_Checklist.docx>. There may be overlap between the checklist items and other queries listed here; please address any duplicated queries both in your response email and on the checklist itself. Upload the completed Human Participants Research checklist as file type “Other” when you re-submit your manuscript. This document is for internal journal use only and will not be published if your article is accepted. The requested information will help us to assess whether your submission complies with PLOS’s policies and adheres to applicable reporting standards. Note that your manuscript may be rejected if you provide incomplete or inadequate responses to the checklist questions and that changing the ‘Section/Category’ of your article does not affect this requirement. | The Human Participants Research checklist has now been uploaded |
| 3. Your study includes live participants, but you have not included an Ethics Statement. Please include an Ethics Statement subsection to your Materials and Methods, to include:  i) The name(s) of the Institutional Review Board(s) or Ethics Committee(s)  ii) The approval number(s), or a statement that approval was granted by the named board(s)  iii) (for human participants or donors) - A statement that formal consent was obtained (must state whether verbal/written) OR the reason consent was not obtained (e.g., anonymity). | This has now been included in the main manuscript and the relevant ethics approval letters uploaded as supporting information. |
| 4. Figure 2: please (a) provide a direct link to the base layer of the map (i.e., the country or region border shape) and ensure this is also included in the figure legend; and (b) provide a link to the terms of use / license information for the base layer image or shapefile. We cannot publish proprietary or copyrighted maps (e.g. Google Maps, Mapquest) and the terms of use for your map base layer must be compatible with our CC-BY 4.0 license.  Note: if you created the map in a software program like R or ArcGIS, please locate and indicate the source of the basemap shapefile onto which data has been plotted.  If your map was obtained from a copyrighted source please amend the figure so that the base map used is from an openly available source. Alternatively, please provide explicit written permission from the copyright holder granting you the right to publish the material under our CC-BY 4.0 license.  Please note that the following CC BY licenses are compatible with PLOS license: CC BY 4.0, CC BY 2.0 and CC BY 3.0, meanwhile such licenses as CC BY-ND 3.0 and others are not compatible due to additional restrictions.  If you are unsure whether you can use a map or not, please do reach out and we will be able to help you. The following websites are good examples of where you can source open access or public domain maps:  * U.S. Geological Survey (USGS) - All maps are in the public domain. (<http://www.usgs.gov>)  * PlaniGlobe - All maps are published under a Creative Commons license so please cite “PlaniGlobe, <http://www.planiglobe.com>, CC BY 2.0” in the image credit after the caption. (<http://www.planiglobe.com/?lang=enl>)  * Natural Earth - All maps are public domain. (<http://www.naturalearthdata.com/about/terms-of-use/>) | The map baselayer was sourced from the Humanitarian Data Exchange and can be found here: https://data.humdata.org/dataset/cod-ab-ukr (Licensed under CC-BY-4.0). This information is now also listed in the Legend of both figure 1 and figure 2.  The terms of use can be found here: https://data.humdata.org/faqs/terms |
