# Supplementary material for: Pre- and post-migration determinants of self-rated health among Ukrainian refugees in Germany: A cross-sectional comparative analysis with recently arrived refugees from other countries of origin
Source: PLOS Glob Public Health. 2025 Nov 10;5(11):e0004565. doi: 10.1371/journal.pgph.0004565 (PMC12599935; doi:10.1371/journal.pgph.0004565)
Supplement: Attachment — Submitted filename: PlosGH_pbyp.docx [file pgph.0004565.s003.docx]

Dear editors and reviewers,

Thank you very much for this opportunity to submit a revised version of our manuscript “Pre- and post-migration determinants of self-rated health among Ukrainian refugees in Germany: a cross-sectional comparative analysis with recently arrived refugees from other countries of origin”. We very much appreciate the detailed and thoughtful comments on our manuscript by the two reviewers. In response, we have substantially revised our manuscript in both form and content and believe that it has been strengthened as a result.

In particular, we have added further details to the introduction and discussion sections contextualising our results in light of the different socio-demographic composition of the two refugee samples studied. We have also added further depth to the discussion by being more specific about our policy options and linking these more explicitly to the results. We have also added to the content of our analysis by performing additional sensitivity analyses, as suggested by Reviewer #2, and are therefore able to give the results of our analysis further depth. A detailed response to each of the reviewers’ comments can be found in the point by point response below (the page numbers refer to the version of the manuscript with tracked changes).

We believe that we were able to address all concerns raised by the reviewers and are therefore looking forward to discussing further steps towards publications.

Kind regards,

The authors

| Reviewer #1: The theme of the article is topical in recent years and this kind of research is needed. Authors used unique datasets on refugees in Germany. I am convinced that the paper presented would be of interest to a wide range of readers. The paper is well written and well structured. However, I have a couple of comments. | Thank you very much for your positive comments and your thoughtful suggestions. |
| --- | --- |
| 1) Could the authors deeper collaborate on differences between Ukrainian and non-Ukrainian refugees and how the characteristics of these two groups could potentially affect the results? The Ukrainian refugees are mostly highly educated women compared to non-Ukrainian refugees mainly men with low education. These are two very different groups of refugees. Ukrainian refugees - women, mostly coming with small children, which may hinder their access to labor market + highly educated women, which may lead to overqualification on the labor market leading to worsening the self-rated health. On the other hand, learning German will be easier for highly educated people. In contrast, non-Ukrainian refugees – men without family obligations, but low educated with less capability to learn German and integrate in the society / labor market. Of course, these different characteristics may lead to different outcomes in terms of self-rated health and their pre- /post-migration determinants. | Thank you for this important suggestion. We have now added further details on the differential socioeconomic composition of our two samples in the introduction (p.3/4, 8/9). We have also included interaction analyses for several important post-migration determinants with gender and education to explore these relationships further and discuss these in the final section of the paper (p.24), see also related comment of Reviewer#2 and our reply. |
| 2) It would be interesting to have some information on the past migration trends, i.e. describe briefly the flow of economic migrants from the given countries (Ukraine, Syria, Iraq, Afghanistan, etc.) before the war conflicts to have an idea about the diaspora network. It was mentioned that lack of contact, isolation, discrimination is a problem. What is the role of diaspora in refugees’ settlement and the consequences on their health outcomes? | Thank you, we have added this to the introduction (p.7) |
| 3) Although the main idea was to compare the Ukrainian refugees and non-Ukrainian refugees in Germany, it would be interesting to have some comparison on the determinants of health among Ukrainian refugees in different countries. For instance, if the results for Germany hold also for Poland, Slovakia, Czechia, Austria etc. Or if the Ukrainian refugees differ by host country. There are some publications on a similar topic that could serve as a basis for brief discussion. - Kohlenberger et al. High self-selection of Ukrainian refugees into Europe: evidence from Krakow and Vienna. Plos One. 2023;18(12):e0279783 - Labberton et al. Trends in the health status of Ukrainina refugees in Norway according to month of arrival during 2022. BMC Public Health. 2024;24(1):3127 - Mazhak et al. Self-reported health and coping strategies of Ukrainian female refugees in the Czech Republic. European Societies. 2024;26(2):411-437 - Kulhánová et al. Determinants of self-rated health among highly educated Ukrainian women refugees in Czechia: analysis based on cross-sectional study in 2022. BMC Women’s Health. 2024;24(1):206 | Thanks for pointing this out and for the additional suggested literature. We have now added several studies on the health of Ukrainian refugees from the European context to the discussion section which provides further international context to the results (p.23/24) |
| 4) Comments related to a formal style of the manuscript: - I guess the authors swapped figure 1 and figure 2. | adjusted |
| - Would it be possible to include the name (or at least the abbreviation) of the Ukrainian regions in the maps in figure 1 and 2? Not all readers have to be familiar with the regional geography of Ukraine. Being able to read the map from your article without going into other literature or internet sources would be helpful. In light of other studies, it would also be helpful for comparison of regions from other publications. | adjusted |
| - In figures 3 and 4, it would be nice to have a clear division of variables belonging to sociodemographic, pre-migration and post-migration factors. I know you described that in the text; however, the figures should be self-explanatory. Drawing a line between the groups of factors would be helpful. | adjusted |
| - Figure 4 should give results for non-Ukrainian refugees, then I do not understand the variable “reason for leaving Ukraine” among pre-migration factors in this regression. Could the authors please explain if this is correct? Or corrected if this was a mistake? | Adjusted – now reads “reason for leaving home country” |
| Reviewer #2: REVIEWER COMMENTS This is an important and interesting study where the authors examined the determinants of self-rated health outcomes amongst Ukrainian refugees in Germany: pre-and-post migration. Authors highlighted that experience of discrimination, and social isolation were critical post-migration factors affecting refugees’ health but those who made attempts to integrate by learning the German language or becoming proficient in the German language have better post-migration health outcomes. These findings are important and highlight the areas in refugee health that might need policy interventions. This work is well written and presented. However, I have few comments for the attention of the authors. | Thank you very much for your encouraging review and the detailed suggestions on the structure and content of our manuscript. |
| INTRODUCTION The introduction provides a solid background, but it could be more concise. Reducing redundancy (e.g., repeated mentions of the EU Temporary Protection Directive) would enhance readability. | Thank you for pointing this out. We agree and have substantially revised the introduction to be more concise. |
| METHODOLOGY The inclusion of the Political Terror Scale (PTS) and war intensity metrics should be better justified and why this threshold of (>3)? Why not use individual-level trauma data? | Thank you. At the time of investigation, occupied Ukrainian territories had been assigned a PTS value of 3, while those not under Russian occupation had a value of 0. This threshold was also applied to the non-Ukrainian sample to aid comparability. Further individual-level data on war experience and trauma were not available in the present study. We have further clarified this on p. 10 of the manuscript. |
| Statistical Analysis I suggest the authors provide more details on missing data patterns and whether MI results differ from complete case analysis. The study uses multiple imputations (MI) to address missing data, which is a valid approach. However, it would be useful to provide more details on: A) missing data patterns-were certain variables missing more frequently? If so, were they missing at random (MAR) or not at random (MNAR)? ; B) Impact of MI-while the Fraction of Missing Information (FMI) was reported (3.5% for Ukrainians, 6.1% for non-Ukrainians), further sensitivity analysis comparing complete case analysis vs. imputed results could add credibility. | We have now added some further details on missing values to the methods section (p. 13). A sensitivity analysis running the models on a non-imputed dataset was already part of the manuscript, with results being reported of p.22/23 and in the Supplementary Information (Tables F and H, last column, sensitivity check SA5). |
| I suggest additional model specifications and fit measures. The reported Variance Inflation Factors (VIFs) suggest no severe collinearity (VIFs range from 1.01–1.72 for Ukrainians, 1.04–1.49 for non-Ukrainians), but it's unclear whether interaction terms were tested. The model fit is evaluated using an F-test (which is appropriate for linear regression but less common for logistic regression). Consider adding: Hosmer-Lemeshow test to check calibration and Pseudo R² (e.g., McFadden’s R²) to assess explanatory power. | Thank you for this suggestion. Unfortunately, more complex tests to assess explanatory power such as the Hosmer-Lemeshow test and Pseudo R2 are not compatible with imputed data, which is why we have made use of the F-test, which is commonly used for imputed models. Apart from that, the interpretation of pseudo R^2 for logit models is not as clear as the interpretation of R^2 in a linear model. Therefore, we refrained from adding this type of measure. |
| I suggest authors should conduct interaction tests for gender, age, and social isolation to check for differential effects. The logistic regression results indicate that post-migration factors (e.g., discrimination, social isolation, language proficiency) are significantly associated with health outcomes in the Ukrainian sample but not in the non-Ukrainian sample (except for social isolation). It’s unclear whether interactions (e.g., between gender and social isolation) were tested. If not, it would be valuable to explore whether certain effects are gender-dependent or vary by age groups. | Thank you very much for this suggestion. We have now conducted additional sensitivity analyses investigating interaction effects between gender and age and three selected post-migration determinants: social interaction, perceived discrimination, language proficiency. Including these interaction terms does not change the key relationships between post-migration factors and health of the main analyses. We discuss the results on p. 23 in our discussion, with full results being presented in the Supplementary Information (Supplementary file, Tables K and L). |
| I suggest stratified analysis by education/employment level to address potential selection bias. The Ukrainian sample is highly educated (70.6% with high education), and 84.4% were employed before flight, which is significantly different from the non-Ukrainian refugee sample (where 60.6% had low education, and only 60.8% were employed pre-migration). Could this education/employment difference bias the health comparisons? A stratified analysis (by education level or employment status) could ensure robustness. | Thank you. We have conducted additional sensitivity analyses stratified by education. This specification does not alter the key relationships between post-migration factors and health compared to the main analyses. We have added this point on p. 23 in our presentation of the sensitivity checks. Full results of the stratifies models are being presented in the Supplementary File (Table K and L). |
| I suggest further sensitivity analysis. The manuscript includes several sensitivity checks, which is a strength. However, the alternative health measure (worries about health) led to some changes in results, suggesting the primary self-rated health measure might not fully capture health conditions. The Political Terror Scale (PTS) vs. war fatalities measure did not show significant associations, but it would be useful to check whether those who fled later in the war (after prolonged exposure) had different health outcomes. | Thank you. We have now conducted an additional sensitivity analysis including the migration cohort (migration before and after June 2022) for the Ukrainian sample. We have added this point on p. 22 in our presentation of the sensitivity checks. Full results of the stratifies models are being presented in the Supplementary File (Table G). |
| DISCUSSION Some arguments could be more explicitly tied to the results. Consider structuring the discussion to first summarize findings, then compare them to existing literature, and finally discuss policy implications. For example, discussing (1) key findings, (2) comparison with prior research, (3) policy relevance. | Thank you, we have made several amendments to the discussion in line with your suggestions, in particular we have tied in the results of our study more strongly, included further references contextualising results in light of the available literature and expanded on the policy implications of our research. |
| It will be valuable to discuss more explicitly the EU Temporary Protection Directive’s limitations and suggest practical steps (e.g., targeted mental health services, language-sensitive healthcare, better social integration strategies). | Thank you, we have substantially expanded on the limitations of the directive and the practical implications for health policy in Germany (p. 24). |
| Consider addressing the “integration paradox” in more depth. Why does early integration not necessarily translate to better health outcomes? How can policies mitigate this? | Thank you. We discuss the idea behind the integration paradox in detail in the section “theoretical background”. We have now anchored it more strongly in the discussion by highlighting the implications for health policy and practice in more detail (p.24) |
| POTENTIAL LIMITATIONS Self-rated health bias: Acknowledge potential cultural differences in self-assessing health and explore how this might affect comparisons between Ukrainian and non-Ukrainian refugees.  Cross-sectional study design: Acknowledge that this study does not establish causality and suggest longitudinal follow-ups to track evolving health conditions. | Both these issues have been addressed in detail in a dedicated paragraph on the limitations of our study (p.25) -> no changes made |
| CONCLUSION The conclusion touches on policy relevance but could be more specific. Suggest concrete measures for improving healthcare access, addressing discrimination, and integrating mental health support. | We have now expanded the section on policy options for responding to the health needs of Ukrainians in Germany (p.24). |
