# Supplementary material for: Pre- and post-migration determinants of self-rated health among Ukrainian refugees in Germany: A cross-sectional comparative analysis with recently arrived refugees from other countries of origin
Source: PLOS Glob Public Health. 2025 Nov 10;5(11):e0004565. doi: 10.1371/journal.pgph.0004565 (PMC12599935; doi:10.1371/journal.pgph.0004565)
Supplement: Attachment — Submitted filename: PlosGH_pbyp_rev2.docx [file pgph.0004565.s004.docx]

Dear editors and reviewers,

We sincerely thank you for the opportunity to resubmit our manuscript in revised form. We have now more explicitly argued the relevance of a comparative analysis of Ukrainian refugees with other refugee groups in Germany. In particular, we have elaborated on both the differences and the meaningfulness of such a comparison, and we have contextualized our findings with regard to their implications for the two refugee groups under study. We have corrected inaccuracies and typographical errors in numerical values throughout the manuscript.

We are convinced that our contribution will have a significant impact on refugee health research. To our knowledge, this is the first study to examine subjective health of two refugee groups who arrived in Germany under very different circumstances and who currently live under very different legal conditions, using representative data. Our findings show that Ukrainians face ongoing difficulties in Germany that are closely tied to health, underscoring the need for accessible healthcare through established models of refugee care. For non-Ukrainian refugees, restrictive legal conditions postpone but do not prevent post-migration health impacts, with barriers to participation posing risks to both individuals and the health system.

We believe that we were able to address all concerns raised by the reviewers and are therefore looking forward to discussing further steps towards publications.

Kind regards,

The authors

| Editor: The short title fails to reflect the comparative nature of the study, which may mislead readers regarding its scope. | Thank you for this advice, we changed the short title accordingly, to “Pre- and Post-Migration Determinants of Health: Ukrainian vs. Other Refugees in Germany”. |
| --- | --- |
| Editor: Furthermore, the rationale for comparing Ukrainian refugees with those from other countries is insufficiently justified, both in the abstract and throughout the paper. This raises concerns about the relevance and interpretability of the analysis, given the substantial demographic and contextual differences between the two groups. | Thank you very much for raising this important point. We have revised the abstract, introduction, and discussion to more clearly emphasize both the relevance and the importance of the comparison between Ukrainian and non-Ukrainian refugees. In particular, we highlight the role of immediate social and health care access granted to Ukrainians under the EU Temporary Protection Directive. This is of high policy relevance, as it allows us to assess its impact on health and to consider whether comparable provisions might be beneficial for other refugee groups without such entitlements.  The comparison also enables us to identify additional determinants of health that may remain masked when access to care is restricted. Importantly, it shows that different legal and reception conditions influence *when and how* post-migration factors shape health outcomes. We now elaborate on these mechanisms in paragraph 2.2. of the theoretical background section. Differences in demographic composition and contextual factors between the two groups are addressed by adjusting for socio-demographic variables in our multivariate models, with corresponding information now added in the methods section.  Finally, we expanded the discussion to make the overall contribution of the present study, and specifically the value of comparing Ukrainian and non-Ukrainian refugees in Germany, more explicit. All changes have been incorporated in tracked-changes mode. |
| Reviewer 2: This is an important and timely manuscript that adds significant value to the literature on refugee health, particularly in the context of the Ukraine crisis. The use of nationally representative datasets, comparative design, and robust statistical methods strengthens the credibility of the findings. The manuscript is well-written, theoretically grounded, and policy-relevant. | Thank you. We are grateful for your positive evaluation of our work. |
| Reviewer 3: The shortened title, “Determinants of health among Ukrainian refugees in Germany,” is clear and concise but does not capture the comparison between the two refugee groups. The short title omits any mention of this comparative aspect, which should be reflected to avoid potential ambiguity about the article’s content. | Thank you for this advice, we changed the short title accordingly, to “Comparing Pre- and Post-Migration Determinants of Health: Ukrainian vs. Other Refugees in Germany”. |
| Reviewer 3: Abstract. The contrast between the two groups is presented in a mechanical rather than a critical manner, and the rationale for comparing refugees from Ukraine with those from other countries is not clearly justified, raising questions about its relevance for publication in a prestigious journal. | Thank you very much for raising this important point, see also our answer #2 to the Editor.  We have revised the abstract, introduction, and discussion to more clearly emphasize both the relevance and the importance of the comparison between Ukrainian and non-Ukrainian refugees. In particular, we highlight the role of immediate social and health care access granted to Ukrainians under the EU Temporary Protection Directive. This is of high policy relevance, as it allows us to assess its impact on health and to consider whether comparable provisions might be beneficial for other refugee groups without such entitlements.  The comparison also enables us to identify additional determinants of health that may remain masked when access to care is restricted. Importantly, it shows that different legal and reception conditions influence *when and how* post-migration factors shape health outcomes. We now elaborate on these mechanisms in paragraph 2.2. of the theoretical background section. Differences in demographic composition and contextual factors between the two groups are addressed by adjusting for socio-demographic variables in our multivariate models, with corresponding information now added in the methods section.  Finally, we expanded the discussion to make the overall contribution of the present study, and specifically the value of comparing Ukrainian and non-Ukrainian refugees in Germany, more explicit. All changes have been incorporated in tracked-changes mode. |
| Reviewer 3: Introduction. I recommend adding a citation to the study by Kulhanova et al. (2022) entitled "Determinants of self-rated health among highly educated Ukrainian refugee women in the Czech Republic: an analysis based on a cross-sectional study in 2022". This study provides an important and timely perspective on the determinants of health within a specific subgroup of Ukrainian refugees - highly educated women - in a Central European context comparable to Germany (34 and 35, p. 4). | We appreciate your suggestion to include this study and have incorporated it into the introduction, with the revisions highlighted in tracked mode. |
| Reviewer 3: p13, line 258, 259: Most Ukrainian refugees have a high level of education (70.6%), but in Table 1 is 73.3% | Thank you for noting this. We have corrected the value in the text to match Table 1 (73.3%), and the change is shown in tracked mode. |
| Reviewer 3: p19, line 264, 265: The proportions by country of origin don't quite match the Table? It should refer to the "country of birth" section. | Thank you for noting this. We re-ran our codes (available online) to re-check the descriptive statistics for both samples presented in Table 1. In doing so, we discovered some incorrectly reported χ² values, probabilities, and proportions (in %), which have now been corrected. All changes are marked in the manuscript in tracked mode.  We also carefully re-checked and revised the descriptions in the text that refer to Table 1, with corrections likewise shown in tracked mode.  Importantly, these corrections do not affect the study’s main results or their interpretation. |
| Reviewer 3: p19, line 265: 57.8% should be 57.9% according to the Table | Thank you for noting this. We have corrected the value in the text to match Table 1 (57.9%), and the change is shown in tracked mode. |
| Reviewer 3: p19, line 268: 75,7 % or 62,2 %? | Thank you for noting this. We have corrected the value in the text to match Table 1 (62.2%), and the change is shown in tracked mode. |
| Reviewer 3: In conclusion, it remains unclear why the comparison between refugees from Ukraine and those from other countries is relevant, given the substantial differences in their legal statuses, migration contexts, and demographic characteristics. The Ukrainian refugee group is relatively homogeneous in terms of nationality, cultural background, timing of arrival, and migration motivation, whereas the non-Ukrainian refugee group is heterogeneous, comprising individuals from diverse countries of origin, cultural backgrounds, lengths of stay, and reasons for migration. These demographic disparities complicate direct comparison and raise questions about the appropriateness and interpretability of the comparative analysis presented. | Thank you for raising this point. We now elaborate more clearly on the benefits of comparing Ukrainian and non-Ukrainian refugees. In particular, we expanded the discussion to provide additional interpretation and to highlight both the necessity of this comparison and the value of our results. All revisions have been made in tracked mode. |
