# Supplementary material for: Pre- and post-migration determinants of self-rated health among Ukrainian refugees in Germany: A cross-sectional comparative analysis with recently arrived refugees from other countries of origin
Source: PLOS Glob Public Health. 2025 Nov 10;5(11):e0004565. doi: 10.1371/journal.pgph.0004565 (PMC12599935; doi:10.1371/journal.pgph.0004565)
Supplement: Attachment — Submitted filename: PlosGH_pbyp_rev3.docx [file pgph.0004565.s005.docx]

Dear Raquel Muñiz-Salazar,

Thank you for giving us the opportunity to revise and improve our manuscript once more. In this revision, we have aimed to describe in detail, within our responses to the individual points raised, the extensions and modifications made in line with the requested changes.

We clarified that existing evidence on refugee health in Germany cannot be extrapolated to Ukrainians, given their distinct demographic, legal, and social circumstances. Direct evidence on their health determinants is therefore needed. By comparing Ukrainian with other refugees, our analysis reveals health trajectories and post-arrival influences, both in unadjusted and adjusted estimates.

Since most existing research on the health of non-Ukrainian refugees focuses on populations several years post-arrival, we contribute by comparing individuals who have been in Germany for one year or less. This short timeframe offers a unique advantage of our dataset and analysis and, to our knowledge, has not been published previously.

We hope that our argumentation in this revision is now convincing and that the added value of our study is clear. We look forward to your feedback.

Sincerely,

Louise Biddle, Andrea Marchitto, Sabine Zinn

| Editor: The current short title is clear but omits the comparative aspect of the study. We recommend revising it to better reflect the analysis of both Ukrainian and non-Ukrainian refugee groups. | Thank you for this advice, we changed the short title accordingly to “Refugee Health in Germany: Comparative Analysis of Ukrainians and Non-Ukrainians”. Now we hope it better reflects the comparative nature of the study. |
| --- | --- |
| Editor: A central concern is the lack of justification for comparing these two groups, given their significant differences in legal status, migration context, and demographics. Clarifying the rationale for this comparison—both in the abstract and introduction—is essential to support the relevance and interpretability of your findings. | Thank you. We have now extensively expanded on our rationale for including a comparison between these two groups in the abstract (lines 2-7), introduction (page 4 lines 47-50, page 5 lines 55-65), methods (page 12 lines 229-230) and discussion (page 23f lines 372-377 & 379-380) of the manuscript. We also discuss the limitations of this comparison (page 24 lines 399-406, page 26f. lines 453-458). Our key point is that, as Reviewer #3 also points out, existing evidence on the health of refugees in Germany cannot be extrapolated to Ukrainians due to different demographic, legal and social circumstances. Thus, direct evidence of their health determinants is needed. Comparing Ukrainian refugees to refugees from other countries of origin helps us to understand the health trajectories and influences in the post-arrival context, both when differences in demographic characteristics are tolerated (unadjusted estimates) and when we take these into account (adjusted estimates). Because existing research on the health of non-Ukrainian refugees is often conducted after several years post-arrival, we provide a comparison of those who have been in the country for one year or less to deepen our understanding of the immediate post-arrival situation for both groups. This short timeframe is a unique benefit of our dataset and our analysis and has not been published previously. |
| Editor: The reviewer also suggests citing Kulhanova et al. (2022), which provides useful context on Ukrainian refugees in a similar European setting. | As requested by Reviewer #3, we have included this reference in our manuscript. It now appears as reference #10 and is cited in the introduction (page 4, lines 39–40).  (Please note that the citation was already present in the original manuscript, but not in the introduction. We have therefore shifted the reference (previously listed as #29) to this earlier point in the text.) |
| Editor: Your statistical approach was well-received, particularly the handling of missing data and sensitivity analyses. However, please ensure consistency between the results reported in the text and the data in Table 1. | Thank you for once more drawing our attention to consistency issues. This careful check revealed two mistakes in the reporting of our statistical findings.  First, we had erroneously stated a significant association between subjective health and pre-flight economic situation and employment status in the non-Ukrainian refugee sample. This was incorrect, and we have now corrected it (page 20, line 301).  Second, we had reported testing interactions of isolation, discrimination, and language proficiency with both gender and education. In fact, the correct variable is age group, not education. We have amended this accordingly (page 13, line 266). |
| Editor: Lastly, we encourage you to address the implications of comparing two demographically distinct groups more explicitly in the discussion and conclusion. | Please see our response to you second point. We have added a discussion of the comparison and its limitations in the discussion section (page 23 lines 372-377, page 24 lines 399-406, page 26f. lines 453-458) of the manuscript. |
